# A nucleotide-sensing endonuclease from the Gabija bacterial defense system

**DOI:** 10.1093/nar/gkab277

**Published:** 2021-04-22

**Authors:** Rui Cheng, Fengtao Huang, Hui Wu, Xuelin Lu, Yan Yan, Bingbing Yu, Xionglue Wang, Bin Zhu

**Affiliations:** Key Laboratory of Molecular Biophysics, the Ministry of Education, College of Life Science and Technology and Shenzhen College, Huazhong University of Science and Technology, Wuhan, Hubei 430074, China; Key Laboratory of Molecular Biophysics, the Ministry of Education, College of Life Science and Technology and Shenzhen College, Huazhong University of Science and Technology, Wuhan, Hubei 430074, China; Key Laboratory of Molecular Biophysics, the Ministry of Education, College of Life Science and Technology and Shenzhen College, Huazhong University of Science and Technology, Wuhan, Hubei 430074, China; Key Laboratory of Molecular Biophysics, the Ministry of Education, College of Life Science and Technology and Shenzhen College, Huazhong University of Science and Technology, Wuhan, Hubei 430074, China; Key Laboratory of Molecular Biophysics, the Ministry of Education, College of Life Science and Technology and Shenzhen College, Huazhong University of Science and Technology, Wuhan, Hubei 430074, China; Key Laboratory of Molecular Biophysics, the Ministry of Education, College of Life Science and Technology and Shenzhen College, Huazhong University of Science and Technology, Wuhan, Hubei 430074, China; Key Laboratory of Molecular Biophysics, the Ministry of Education, College of Life Science and Technology and Shenzhen College, Huazhong University of Science and Technology, Wuhan, Hubei 430074, China; Key Laboratory of Molecular Biophysics, the Ministry of Education, College of Life Science and Technology and Shenzhen College, Huazhong University of Science and Technology, Wuhan, Hubei 430074, China

## Abstract

The arms race between bacteria and phages has led to the development of exquisite bacterial defense systems including a number of uncharacterized systems distinct from the well-known restriction-modification and CRISPR/Cas systems. Here, we report functional analyses of the GajA protein from the newly predicted Gabija system. The GajA protein is revealed as a sequence-specific DNA nicking endonuclease unique in that its activity is strictly regulated by nucleotide concentration. NTP and dNTP at physiological concentrations can fully inhibit the robust DNA cleavage activity of GajA. Interestingly, the nucleotide inhibition is mediated by an ATPase-like domain, which usually hydrolyzes ATP to stimulate the DNA cleavage when associated with other nucleases. These features suggest a mechanism of the Gabija defense in which an endonuclease activity is suppressed under normal conditions, while it is activated by the depletion of NTP and dNTP upon the replication and transcription of invading phages. This work highlights a concise strategy to utilize a DNA nicking endonuclease for phage resistance via nucleotide regulation.

## INTRODUCTION

To resist frequent and diverse attacks by bacteriophages, bacteria have developed multiple, exquisite defense strategies that can collectively be referred to as the bacterial ‘immune system’ (1–3). Anti-phage defense strategies include the adaptive immune system CRISPR/Cas, which provides acquired immunity by memorizing past phage invasion (4); innate immune restriction-modification (R-M) systems that target specific sequences in viral DNA (5); abortive infection (Abi) systems that cause cell death or metabolic disturbance upon phage infection (6); and additional systems with mechanisms that are not yet clear. In recent years, CRISPR/Cas9 gene editing technology derived from the CRISPR/Cas system has been developed swiftly and is now the most widely used gene editing method. Similarly, restriction endonucleases derived from R-M systems previously led to revolutions in recombinant DNA technology and serve as key enzymatic reagents for modern molecular biology. The most widely used restriction enzymes in the laboratory are Type II restriction endonucleases, which are further classified into 12 subtypes: A, B, C, E, F, G, H, L, M, P, S and T based on their properties and behavior (7–9). For example, the enzymes of the ‘IIP’ subtype recognize palindromic (symmetric) DNA sequences and the ‘IIS’ subtype enzymes are characterized by shifted cleavage. Pingoud *et al.* have discussed the mechanisms of sequence recognition and catalysis of Type II restriction endonucleases systematically (10).

The recent boom in metagenomic analyses has suggested that a large number of uncharacterized defense systems exist in bacteria (11). As predicted, an increasing number of defense systems have been validated successively (12–14). Doron *et al.* predicted and experimentally verified 10 potential anti-phage defense systems, although their molecular mechanisms are not yet understood (12). Recent progress on the underlying mechanism of the Thoeris defense system implies that NAD+ degradation is a unique strategy for bacterial anti-phage resistance (15,16).

Among these newfound systems, we focus on the Gabija bacterial defense system, which contains two components, GajA and GajB. As shown in a previous study, the Gabija system from *Bacillus cereus* VD045 shows potent defense against bacteriophages phi29, rho14, phi105 and SpBeta (12). Bioinformatic analysis suggests that the Gabija system is widely distributed in bacteria and archaea and exists in at least 8.5% of sequenced genomes that have been analyzed (4360 genomes) (12). In comparison, CRISPR/Cas systems are found in about 40% of all sequenced bacteria (17,18), R-M systems are found in about 75% of prokaryote genomes (19), and prokaryotic Argonautes (pAgos) and Bacteriophage Exclusion (BREX) systems appear in about 10% of sequenced prokaryote genomes (20–22). Many known bacterial defense systems attack bacteriophage genomic DNA and most of their elements have the ability for specific nucleic acid processing, such as CRISPR/Cas and R-M systems (4,5). In this study, we elucidated the function of the GajA protein, which consists of an N-terminal ATPase-like domain and a C-terminal TOPRIM domain, and has been predicted to be an ATP-dependent nuclease. Among characterized nucleases, the recently reported nonspecific nucleases of the overcoming lysogenization defect (OLD) family involved in DNA repair and replication, including BpOLD, XccOLD and TsOLD (23,24), share the highest homology with GajA. GajB has been predicted to be a UvrD-like helicase (12).

In this work, we purified the GajA protein from *B. cereus* VD045 and characterized its function. We found that GajA exhibits specific DNA nicking endonuclease activity. We defined the cleavage site, recognition sequences, optimal reaction conditions and functional domains of GajA. We further revealed that GajA activity is negatively regulated by nucleotides, and that the H320A mutation in the ATPase-like domain partially relieves the ATP inhibition of GajA cleavage activity. Overall, we demonstrated that GajA is a novel nucleotide-sensing nicking endonuclease and proposed a novel strategy relying on nucleotide regulation for anti-phage resistance as part of the molecular mechanism underlying the Gabija bacterial defense system.

## MATERIALS AND METHODS

### Materials

Oligonucleotides and primers were obtained from Genscript Company. The Gibson assembly kit, Quick Blunting™ Kit, alkaline phosphatase and T4 DNA ligase were from New England BioLabs. T-plasmid (catalog no. C601) was from Vazyme Biotech. PrimeSTAR Max DNA Polymerase was from TaKaRa, and the DNA purification kit was from Axygen. Ni-NTA resin was from Qiagen. Preparative Superdex S200 (catalog no. 17-1043-01) for gel filtration was from GE Healthcare. ATPase activity was quantified using the PiColorLock™ phosphate detection system kit (Expedeon). DNA marker (#SM0331) and protein marker (#26619) were from Thermo Scientific™.

### Cloning, expression and purification of GajA

The predicted coding sequence (AHET01000033.1: 94 190–95 926, and the whole sequence listed in Supplementary Table S1) for GajA (residues 1–578), its active site mutants, and GajA-CTR (residues 348–578) were cloned into pET28a vectors (between Nde I and Not I sites) harboring an N-terminal 6 × His tag using Gibson Assembly Cloning Technology (25). Constructs were transformed into *Escherichia coli* BL21(DE3) cells, which were cultured in 2 l LB medium containing 50 μg/ml kanamycin at 37°C for 3 h to an OD_600_ of 0.6–0.7, and then induced with 0.1 mM IPTG for 18 h at 12°C.

The cells were harvested and resuspended in lysis buffer (20 mM Tris–HCl, pH 7.5 at 25°C, 300 mM NaCl and 0.5 mM DTT), then lysed by ultrasonication. Supernatant was collected after centrifugation for 1 h at 20 000 × g, 4°C and filtrated with 0.45-μm filter. The filtered supernatant was loaded onto a Ni-NTA agarose column pre-equilibrated with 10 volumes of elution buffer (20 mM Tris–HCl pH 7.5, 300 mM NaCl), and then the column was washed with 10 volumes of elution buffer containing 20 mM and 50 mM imidazole, respectively. The majority of GajA was eluted by elution buffer containing 100 mM imidazole. Collected eluates were concentrated to 2.5–3 ml by Millipore Amicon Ultra-15 (30 000 MWCO) and further purified by gel filtration chromatography on a 200-ml preparative Superdex S200 column. Fractions containing pure GajA were concentrated again. Finally, GajA was dialyzed against a storage buffer containing 50 mM Tris–HCl pH 7.5, 100 mM NaCl, 1 mM DTT, 0.1 mM EDTA, 50% glycerol and 0.1% Triton X-100. Protein concentrations of GajA were determined using a Bradford protein quantitative kit (Bio-Rad), and protein purity and concentration were analyzed by 10% SDS-PAGE stained with Coomassie blue (Bio-Rad).

Active site mutations and GajA-CTR were introduced via the Gibson Assembly method (25) (primers for cloning are listed in Supplementary Table S2), and mutants were expressed and purified with the same procedure as detailed above.

### DNA cleavage assays

To explore the requirement of GajA for metal ions, DNA cleavage experiments were carried out at 37°C in reaction buffer (20 mM Tris–HCl, pH 9 and 0.1 mg**/**ml BSA) supplemented with 5 mM MgCl_2_, MnCl_2_, CaCl_2_, ZnCl_2_, CoCl_2_ or NiCl_2_. After screening the optimal reaction conditions, 125 ng of DNA substrate (20 nM) was incubated with 0.2 μM protein in a final volume of 10 μl in DNA cleavage buffer (20 mM Tris–HCl, pH 9, 1 mM MgCl_2_ and 0.1 mg**/**ml BSA). Reactions were performed at 37°C for 2 or 5 min and then stopped by the addition of 2 μl of 6× loading dye containing 20 mM EDTA. Samples were analyzed via native agarose gel electrophoresis. After ethidium bromide staining, the signal of the initial DNA substrate was measured and quantified using ImageJ software (26). To determine the ratio of degraded DNA to intact DNA, the intensity of the intact DNA substrate band in each gel lane was compared with the intensity of the intact DNA band in the protein-free control lane. The quantification bar graphs represent the average of three independent trials with error bars representing the standard error of the mean.

### Determination of the DNA cleavage site

A 955-nt DNA fragment derived from lambda phage genomic DNA (λ955) located at 1985–2939 was cut into two small fragments, λ372 and λ583, by GajA. The latter two fragments were recovered, blunt ends were created using the Quick Blunting™ Kit, and fragments were respectively inserted into T-plasmids, respectively. Several colonies each were selected for DNA sequencing, and the GajA cleavage site was deduced from the sequencing results.

### Determination of the recognition sequence

In order to identify the full recognition sequence of GajA, we inserted the DNA sequences surrounding the GajA DNA cleavage site into pUC19 plasmid and introduced various sequence alterations into the inserted region using the Gibson Assembly method. The constructed plasmids were verified by DNA sequencing, and DNA fragments for GajA cleavage substrates were amplified by PCR using primers pUC19-F/R (Supplementary Table S3).

### Synthetic DNA substrates

The synthetic DNA substrate was prepared by mixing equimolar amounts (20 μM) of complementary 56-nt oligonucleotides in a total volume of 20 μl of annealing buffer (10 mM Tris–HCl pH 7.4, 50 mM NaCl). Complementary oligonucleotides were annealed by heating at 95°C for 5 min and then gradient cooling to room temperature over a 100-min period. In the oligoduplex cleavage assay, the reaction mixtures containing 0.4 μM GajA and 0.8 μM DNA were incubated at 37°C for 2 min. The reactions were stopped by the addition of the loading dye containing 20 mM EDTA and analyzed by 12% PAGE.

### DNA nicking assays

DNA nicking assays were carried out on various DNA fragments covering bacteriophage T7 genomic DNA in 200 μl volume at 37°C for 5 min. DNA nicking reactions contained 20 mM Tris–HCl (pH 9), 1 mM MgCl_2_, 1 mM DTT, 20 ng/μl PCR-amplified DNA substrates and 0.2 μM GajA. After reactions, products were recycled. DNA nicking sites were determined by run-off Sanger sequencing as previously described (27,28). The nicking site consensus sequences were compiled with WebLogo server (https://weblogo.berkeley.edu) (29).

### ATPase assays

ATPase activity was measured using analysis of the products of cleavage by thin layer chromatography. The reaction was carried out in ATPase reaction buffer (20 mM Tris-OAc pH 7.9, 50 mM K-OAc, 10 mM Mg-OAc, 1 mM DTT) with 4 mM ATP, 12 μM 56-bp DNA (DNA fragment S1 used in Figure 2C) and 3 μM or 6 μM protein at 37°C. After 60-min incubation, 1 μl samples were spotted onto a polyethyleneimine cellulose TLC plate and developed with a solution containing 1 M formic acid and 0.8 M LiCl as previously described (30).

ATPase activity was quantified using a phosphate detection system kit that monitored the amount of free phosphate released. The reactions were performed in ATPase reaction buffer as aforementioned with 0.5 mM ATP, 2 μM 56-bp DNA (DNA fragment S1 used in Figure 2C), and 1 μM protein for 1 h at 37°C. Subsequent processing was carried out according to the kit manual and samples were measured by a NanoPhotometer^®^ (Implen) at 650 nm.

## RESULTS

### GajA exhibits specific cleavage activity *in vitro*

**Figure 1.**
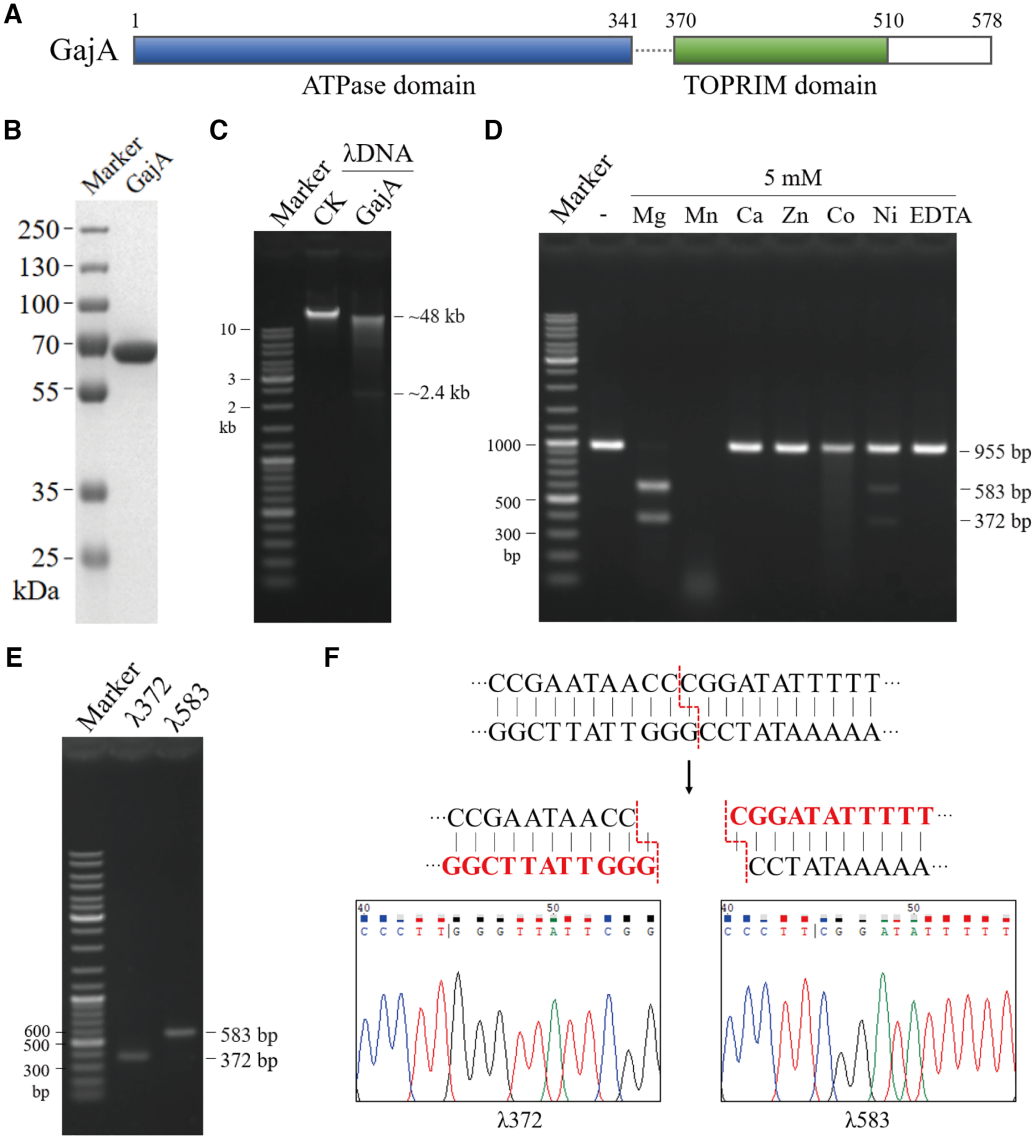
Purified GajA as an endonuclease. (**A**) Domain architecture of GajA protein. (**B**) SDS-PAGE gel showing purified GajA (69 kDa including an N-terminal His-tag). (**C**) Cleavage of linear λDNA by GajA. (**D**) GajA nuclease activity is dependent on metal ions. Reaction mixtures containing 20 mM Tris–HCl pH 9, 0.1 mg**/**ml BSA, 20 nM λ955 DNA, 200 nM GajA and 5 mM metal ions (MgCl_2_, MnCl_2_, CaCl_2_, ZnCl_2_, CoCl_2_ or NiCl_2_ as shown on top of the gel) were incubated at 37°C for 5 min. Gel bands corresponding to the 955-bp λ955 DNA substrate and 583-bp and 372-bp DNA products resulting from specific endonuclease cleavage are annotated. Reactions with no metal ions added (-) and 5 mM EDTA were included as controls. (**E**) Recovery of the two DNA fragments (λ372 and λ583) from GajA endonuclease cleavage for cloning and sequencing. (**F**) The GajA cleavage site and pattern were determined based on DNA sequencing. The λ372 and λ583 fragments were inserted into T-plasmids and the regions near the cleavage site were respectively sequenced. Red sequences are the terminal sequences of each fragment derived from the sequencing results (bottom), and the dotted red lines demonstrate the cleavage site.

The Gabija system exists in about 8.5% of all sequenced bacteria and archaea (12). It consists of two components, GajA and GajB (12,23,24). Bioinformatic analysis indicated that GajA contains an ATPase-like domain (residues 1–341) and a TOPRIM domain (residues 370–510) (Figure 1A). As the major element in the Gabija system for phage resistance, GajA was initially suspected to function as a nuclease. To elucidate its function, GajA with over 90% homogeneity was purified as an N-terminal His-tagged protein (Figure 1B). The potential nuclease activity of GajA was tested on various nucleic acid substrates and among random DNA and RNA substrates, such as pUC19 plasmid, λDNA, M13 ssDNA, and sox7 RNA (the sox7 RNA sequence is listed in Supplementary Table S4), but specific dsDNA cleavage activity was detected only on λDNA. A specific fragment of about 2.4 kb was produced when λDNA was treated by GajA (Figure 1C). Subsequently, λDNA was divided into eight segments by PCR amplification, among which only the 5′ foremost 6 kb region of λDNA was cleaved by GajA into a 2.4 and 3.6 kb fragment (Supplementary Figure S1A). This substrate was further shortened to 955 bp, which was cut into a 583 bp (λ583) and a 372 bp (λ372) fragment by GajA (Supplementary Figure S1B).

With λ955 DNA as a substrate, we first examined the effect of divalent cations on the efficiency and specificity of GajA (Figure 1D). Cleavage efficiency was quantified by comparing the band intensity in each lane and calculating the percentage of DNA digested relative to the control. At the same divalent cation concentration of 5 mM, GajA exhibited rapid specific cleavage in the presence of Mg^2+^, degrading approximately 100% of the substrate within 5 min. In the presence of Mn^2+^, GajA degraded the DNA substrates into small pieces without showing specificity. Weak but specific GajA activity was observed in the presence of Ni^2+^, while weak and nonspecific cleavage was shown in the presence of Co^2+^. Ca^2+^ and Zn^2+^ had no prominent influence on GajA activity (Figure 1D). These data indicate that metal ions are required for GajA activity, and Mg^2+^ is optimal for the specific DNA cleavage of GajA. Thus, the λ583 and λ372 fragments resulting from the specific cleavage by GajA in the presence of Mg^2+^ were purified (Figure 1E) and respectively cloned into T-plasmids to reveal the cleavage site of GajA. Fifteen clones each were selected for DNA sequencing and the results were uniform, and through them the GajA cleavage site was deduced (Figure 1F). Apparently, the cleavage site is not located in a typical palindromic sequence as those recognized by Type IIP restriction enzymes.

### Optimal reaction conditions of GajA

Before investigation of the GajA recognition sequence, we optimized the reaction conditions including the concentration of divalent metal ions, pH, and temperature for GajA activity. GajA cleavage activity was optimal in the presence of 1–5 mM Mg^2+^ and decreased at higher or lower concentrations of Mg^2+^ (Supplementary Figure S2A). GajA exhibited specific DNA cleavage at low concentrations of Mn^2+^ (Supplementary Figure S2B), but with [Mn^2+^] higher than 20 μM nonspecific cleavage appeared. Calcium did not potentiate but rather inhibited GajA nuclease activity when supplied with magnesium (Supplementary Figure S2C). At physiological concentrations (31,32), both Mg^2+^ (4–5 mM) and Mn^2+^ (∼15 μM) supported the specific endonuclease activity of GajA, while Co^2+^ and Ni^2+^ (∼0.5 mM) inhibited GajA activity (Supplementary Figure S3). In 20 mM Tris–HCl, GajA activity was most efficient at pH 9 (Supplementary Figure S4A) and at 37°C or 42°C (Supplementary Figure S4B). GajA is sensitive to salt, as 100 mM NaCl or KCl completely inhibited its activity (Supplementary Figure S5). Thus, we have established the optimal reaction conditions for GajA as 20 mM Tris–HCl pH 9, 1 mM MgCl_2_, and 1 mM DTT, at 37°C.

λ955 DNA fragments, whether prepared by PCR amplification or directly extracted from plasmids, were cleaved by GajA with similar efficiency (Supplementary Figure S6). GajA also cut the ‘supercoiled’ pUC19 plasmid containing the λ955 sequence into ‘linearized’ and ‘nicked’ DNA (Supplementary Figure S6). GajA exhibited no nuclease activity on single-stranded DNA (ssDNA), double-stranded RNA (dsRNA), or single-stranded RNA (Supplementary Figure S7) (all substrate sequences are listed in Supplementary Table S5, and all substrates contain the recognition sequence of GajA [underlined in Supplementary Table S5]). These data suggest that GajA cuts double-stranded DNA (dsDNA) specifically in a sequence-dependent manner.

### Recognition sequence of GajA

**Figure 2.**
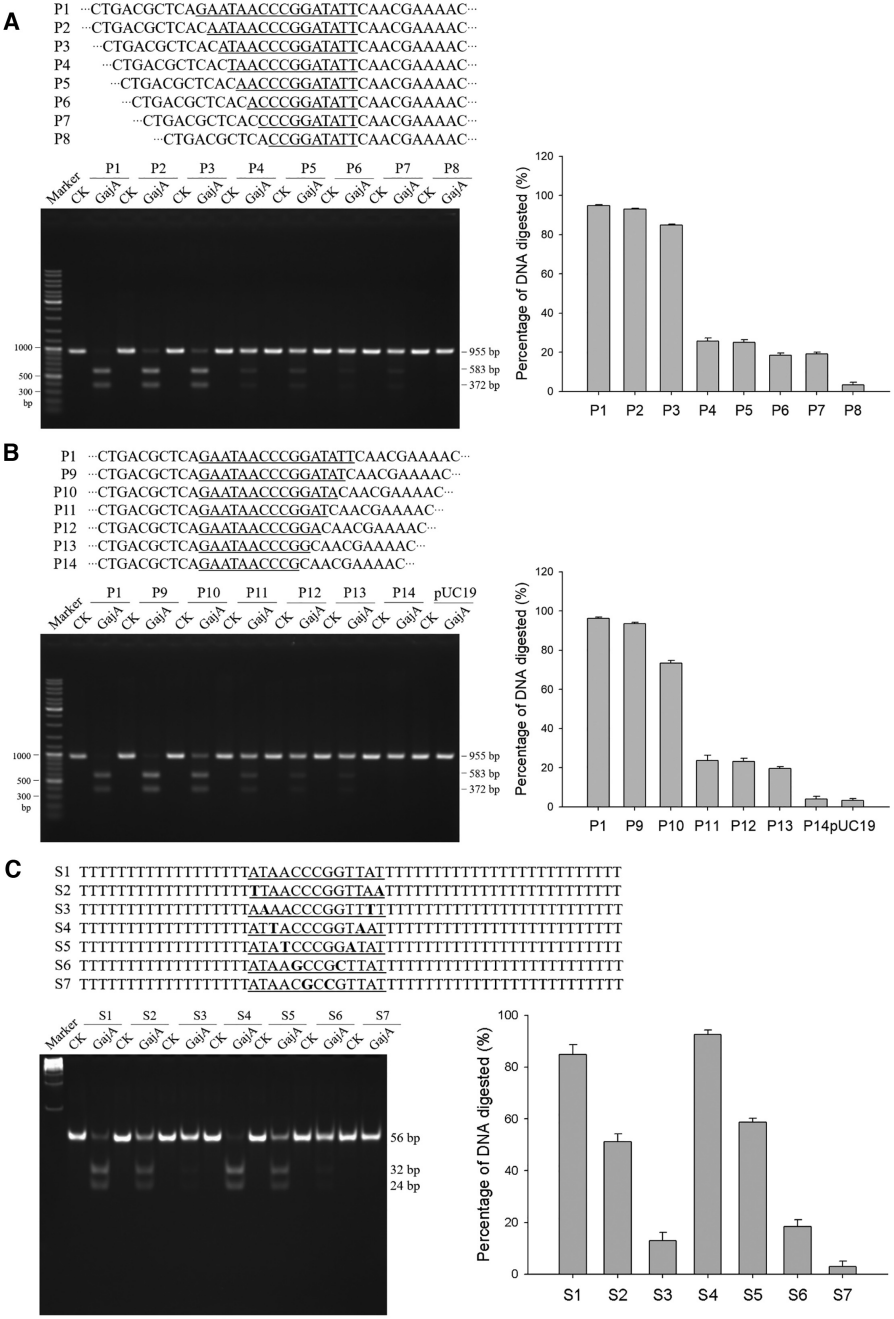
Characterization of the full optimal recognition sequence of GajA. The preliminary recognition sequence of GajA was shortened one nucleotide at a time from the left end (**A**) or right end (**B**), and DNA fragments containing the resulting sequences were PCR-amplified to be used as substrates for GajA. (**C**) Cleavage efficiency (measured as a reduction of initial DNA substrates) of GajA on DNA oligos with base-switching in the palindromic region. The synthetic DNA substrates were prepared by mixing equimolar amounts of complementary 56-nt oligonucleotides. The DNA substrate (800 nM) containing one GajA recognition site was digested by GajA (400 nM), and results were analyzed by 10% PAGE. DNA digestion was measured using ImageJ software as described in the Materials and Methods. All graphs represent the average of three independent trials with error bars representing the standard error of the mean.

The λ955 DNA fragment was cut into a 583-bp (λ583) and a 372-bp (λ372) fragment by GajA (Supplementary Figure S1B). However, neither of the T-plasmids inserted with λ583 or λ372 were linearized by GajA, suggesting that sequences on both sides of the cleavage site are required for GajA recognition. Therefore, we focused on the sequences surrounding the cleavage site in λ955 and gradually shortened it to the minimum GajA recognition sequence. Various recognition sequences were inserted into the pUC19 plasmid and then amplified by common primers to be tested as GajA substrates. First, we found that a the 16-bp sequence 5′-GAATAACCCGGATATT-3′ containing the cleavage site is sufficient for GajA recognition (Supplementary Figure S8). With gradual one-by-one nucleotide shortening on either side of the 16-bp sequence, GajA cleavage efficiency gradually decreased correspondingly (Figure 2A and B) until reaching into the center CCCGG sequence, for which a single nucleotide deletion on either side abolished GajA activity. Meanwhile, truncations on both ends of the 16-bp sequence simultaneously caused a rapid decline of GajA cleavage efficiency (Supplementary Figure S9). From these results, it seemed that the center 5-bp GC-rich core sequence in combination with the 5-bp AT-rich wing sequence on either side (AATAACCCGG or CCCGGATATT) is a minimum recognition sequence to maintain GajA cleavage, while the overlapping of these two minimum recognition sequences constitutes the full 15-bp restriction site for GajA. Interestingly, we noticed that in the 15-bp GajA restriction sequence (5′-AATAACC*C*GG**A**TATT-3′) as deduced above, the 7-bp sequences on either side of the center deoxycytidine (italic) are nearly palindromic. An A (bold)-to-T mutation to perfect the palindrome resulted in the most efficient GajA restriction sequence (5′-AATAACC*C*GG**T**TATT-3′) (Supplementary Figure S10).

We examined the degeneration of the GajA recognition sequence. Either a G addition or a C deletion in the core region to make the whole sequence palindromic reduced the cleavage efficiency (Supplementary Figure S11). Conversion of the center C to T only decreased the cleavage slightly (and the effect of G or A was concurrently examined at the equivalent position in the complementary DNA strand); thus the center nucleotide can be any of the four bases (Supplementary Figure S11A). We also switched each pair of nucleotides in the palindromic region and found that all the GC-rich core sequence (except for the center nucleotide) and the middle nucleotide of the AT-rich wing sequence are more crucial for GajA recognition, as alterations of these nucleotides decreased the GajA cleavage activity more severely than those of other positions (Figure 2C). Altogether, we uncovered the full optimal recognition sequence and cleavage pattern for GajA as a sequence-specific endonuclease (Supplementary Figure S11B).

### Turnover of GajA on DNA substrates

**Figure 3.**
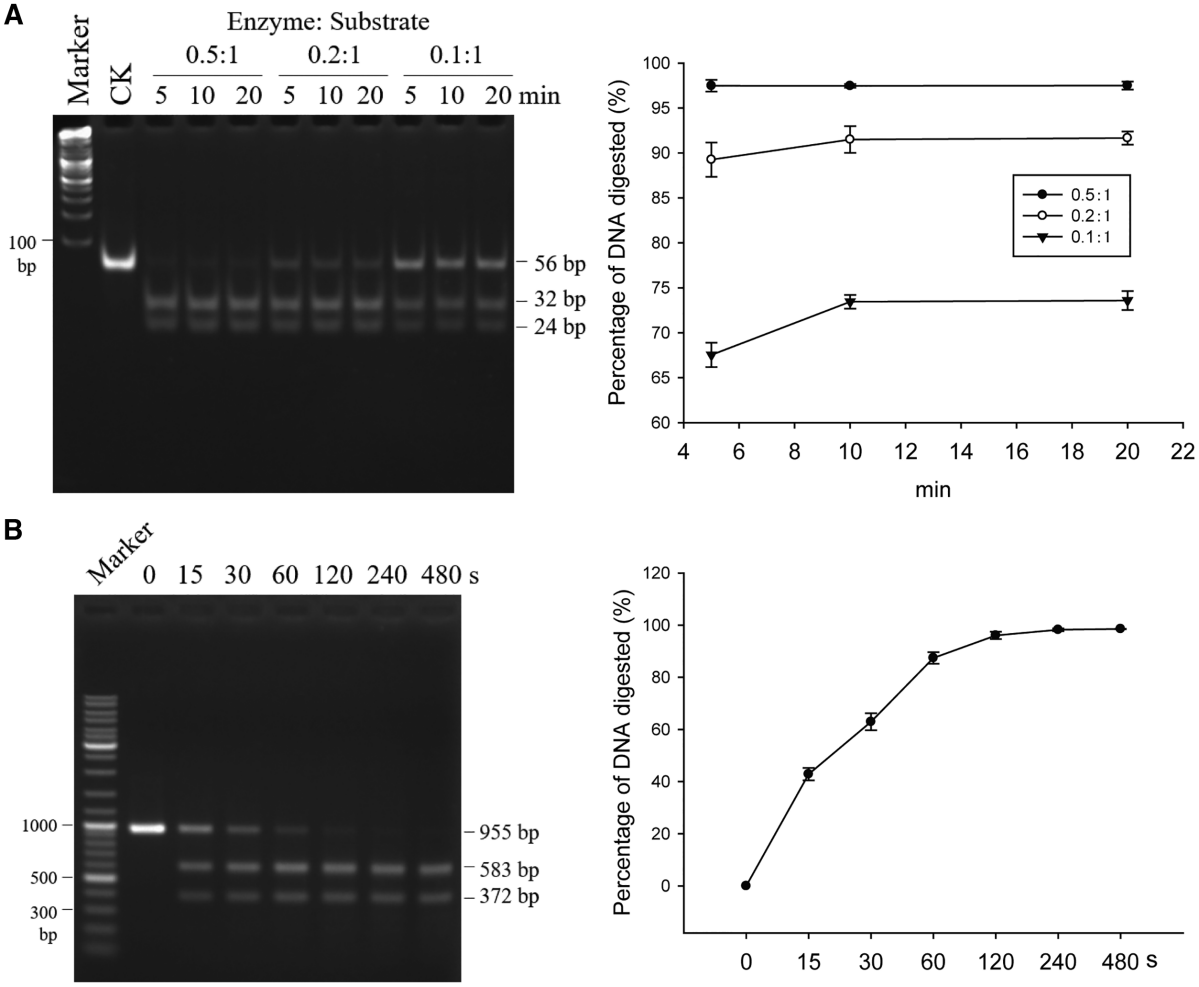
Turnover and efficiency of GajA. (**A**) Cleavage efficiency of GajA at different molar ratios of enzyme to substrate. The synthetic DNA oligo S1 (0.8 μM) was incubated with GajA (0.4 μM) and the reaction time was set as 5, 10, or 20 min, respectively. (**B**) GajA exhibits rapid DNA cleavage activity. λ955 DNA was used as the substrate. Over 60% of the DNA substrate (20 nM) was digested by GajA (200 nM) after 30 s, and over 96% was digested after 120 s. Reactions were performed in a final volume of 10 μl in the optimal reaction buffer at 37°C and then stopped by addition of 2 μl of 6× loading dye containing 20 mM EDTA. Samples were analyzed via native agarose gel electrophoresis. All graphs represent the average of three independent trials with error bars representing the standard error of the mean.

A synthetic DNA substrate (S1 fragment in Figure 2C) was incubated with diluted GajA for 5, 10 and 20 min. With excess DNA substrate, decreasing the molar ratio of GajA to substrate resulted in less DNA cleavage, and an extension of the reaction time from 5 to 20 min did not increase the cleavage significantly (Figure 3A), indicating that the turnover of GajA is inefficient. However, when enzyme was in excess (20 nM DNA substrate was digested by 200 nM GajA), the GajA exhibited rapid DNA cleavage activity, as over 60% of the initial DNA substrate was digested after 30 s, and over 96% of the DNA substrate was digested after 120 s (Figure 3B).

### DNA nicking by GajA

**Figure 4.**
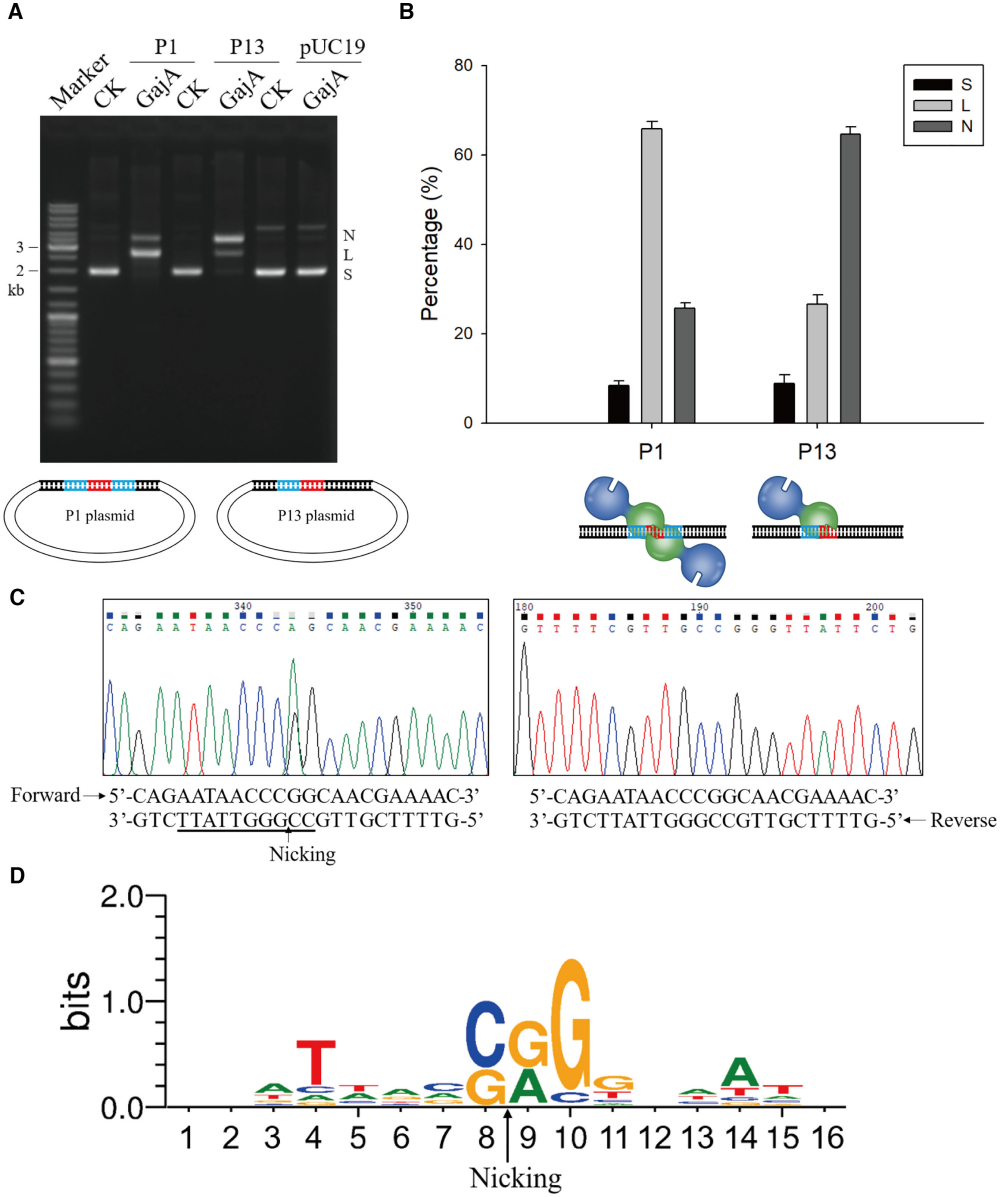
GajA is a site-specific nicking enzyme. (**A**) GajA cleavage patterns on various plasmids. The P1 plasmid contains the complete GajA restriction sequence consisting of two overlapping minimum recognition sequences, P13 contains one minimum recognition sequence, and pUC19 without the GajA recognition sequence was used as a control. The sequence of the colored DNA region in P1 is 5′-AATAACCCGGATATT-3′ and that in P13 is 5′-AATAACCCGG-3′. ‘N’, ‘L’, and ‘S’ denote the positions of gel bands corresponding to ‘nicked’, ‘linearized’, and ‘supercoiled’ DNA, respectively. In the plasmid diagram, the core GC-rich region of the GajA recognition sequence is shown in red and the AT-rich wing region in blue. (**B**) Quantification of products of GajA cleavage on plasmids P1 and P13. Proportions of nicked, linearized, and supercoiled DNA after GajA treatment were compared. The bottom diagram depicts the two action modes of GajA endonuclease. (**C**) Nicking site of nicked P13 plasmid after incubation with GajA. The nicked DNA of P13 plasmid after incubation with GajA was recycled separately and subjected to DNA sequencing. The overlapping double peak indicates the nicked site (denoted by the arrow). The forward sequencing result exhibited an additional peak corresponding to ‘A’, while the reverse sequencing was normal. (**D**) The nicking sites of GajA in six fragments of T7 genomic DNA compiled by the WebLogo server. The overall height of each stack indicates the sequence conservation at that position (measured in bits), and the height of symbols within the stack reflects the relative frequency of the corresponding base at that position. The arrow indicates nicking site.

The optimal GajA recognition sequence (5′-AATAACCNGGTTATT-3′) consists of two overlapping (5′-AATAACCNGG-3′) sequences of opposite orientation (Supplementary Figure S11B). The above results showed that disruption in one of the two overlapping sequences did not abolish the cleavage by GajA (Figure 2A and B), indicating that each of the 5′-AATAACCNGG-3′ sequences may function independently to trigger DNA nicking activity. Because a single nicking event in linear dsDNA is not easy to observe, we constructed plasmids P1 and P13. P1 contains the intact GajA restriction sequence and therefore the suspected nicking recognition sequence, 5′-AATAACCNGG-3′, is presented in both DNA strands and orientations, while P13 contains only a single sequence 5′-AATAACCNGG-3′ (Figure 4A). After incubation with GajA, the majority of supercoiled P1 plasmid was cleaved into linear DNA, with a small portion of nicked plasmid. In contrast, most of the P13 plasmid was turned into nicked DNA (Figure 4A and B). The overall cleaved portion for both plasmids was similar. These results confirmed that the GajA is a nicking endonuclease, and the 5′-AATAACCNGG-3′ sequence is sufficient to trigger its DNA nicking activity. In order to demonstrate the exact GajA nicking site, the nicked DNA of the P13 plasmid after incubation with GajA was gel-purified and subjected to run-off sequencing. The forward sequencing result exhibited an additional peak corresponding to ‘A’ (the Taq DNA polymerase adds an additional A at the 3′ terminus of the newly synthesized DNA strand upon the 5′ terminus of the template DNA strand during sequencing reactions), while the reverse sequencing result was normal (Figure 4C). These results demonstrated that the minus strand as shown in Figure 4C is nicked (5′-CC↓GGGTTATT-3′; the down arrow marks the nicking site). The unique nicking recognition sequence gives GajA functional flexibility, as the previously observed restriction enzyme-like dsDNA cleavage activity of GajA is due to the arrangement of two overlapping nicking recognition sequences at both DNA strands (Supplementary Figure S11B).

As GajA is a sequence-specific DNA nicking enzyme, our previous assays focusing on the detection of dsDNA breaks might have missed the native GajA nicking sequences. Thus, we extensively investigated the GajA nicking sequences on its native DNA substrates—the genomic DNA from bacteriophages that the Gabija system can resist. To this end, the Gabija gene cassette containing GajA and GajB genes from *B. cereus* VD045 was cloned into plasmid pQE82L and introduced into *E. coli* B (ATCC^®^ 11303™). The expression of the *B. cereus* VD045 Gabija gene cassette gave the E. coli strain strong resistance to bacteriophage T7, as the phage infection efficiency dropped more than 10^6^-fold (Supplementary Figure S12A). To reveal the nicking sites of GajA on T7 genomic DNA, the 39937-bp T7 genomic DNA was divided into six segments by PCR amplification, and these fragments were respectively treated by GajA (Supplementary Figure S12B). After incubation with GajA at 37°C for 5 min, the DNA substrates were subjected to extensive run-off sequencing covering the whole T7 genome from both orientations. Judging from the additional ‘A’ peaks in the sequencing peak maps similar to that shown Figure 4C, 110 nicking sites were identified and the 8-nt sequences on both sides of the nicking sites are listed in Supplementary Table S6. Several examples of the sequencing results revealing GajA nicking sites are shown in Supplementary Figure S13. The frequencies of each of the four bases at each position of the 8-nt upstream and downstream sequences of the 110 nicking sites were calculated and are shown in Figure 4D. If the frequency of a base at a given position was more than 0.2 bits (the overall height of each stack proportional to the sequence conservation, measured in bits), the base was considered as a preferred base for GajA recognition. Following this standard, the GajA nicking recognition sequence was deduced as 5′-TNNNS↓RGGNNA-3′ (DNA single letter code, S: G/C; R: A/G; N: A/G/C/T), and the optimal nicking recognition sequence was 5′-TNNNC↓GGGNNA-3′, which was generally consistent with our previously identified optimal recognition sequence 5′-CC↓GGGTTATT-3′ through in vitro mutagenesis (Figure 4C and Supplementary Figure S11B).

### Cleavage activity of GajA is suppressed by nucleotides

**Figure 5.**
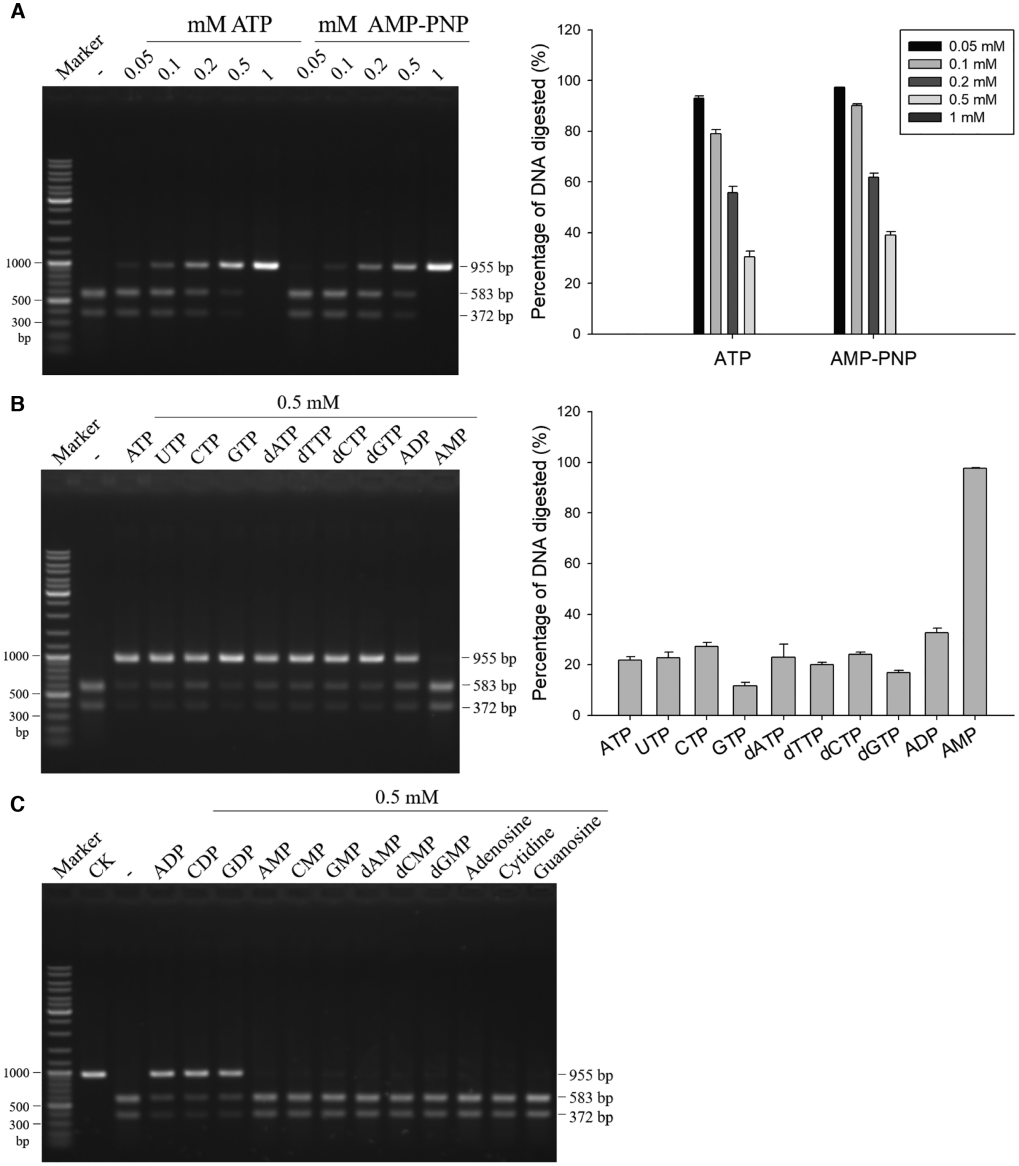
The endonuclease activity of GajA is inhibited by nucleotides. (**A**) Representative gel and quantification of GajA endonuclease activity on λ955 DNA in the presence of increasing amounts of ATP and AMP-PNP. (**B**) Effect of NTP, dNTP, ADP, and AMP on GajA endonuclease activity. (**C**) Effect of NDP, NMP, dNMP and nucleosides on GajA endonuclease activity. All reactions contained 20 nM λ955 DNA and 200 nM GajA and were incubated at 37°C for 5 min. Lanes labeled with dashes indicate no nucleotide addition. Initial DNA digested was quantified using ImageJ software. Bar graphs represent the average of three independent experiments with error bars representing the standard error of the mean.

An N-terminal ATPase-like domain occupies more than a half of the GajA polypeptide (Figure 1A), naturally raising the possibility that ATP hydrolysis stimulates the endonuclease activity as reported previously for the MLH1–MLH3 complex (33). However, thin layer chromatography ATPase assay revealed that GajA has no ATPase activity, which was further confirmed by monitoring the amount of free phosphate released (Supplementary Figure S14A and B). When we added ATP into the GajA reaction to test whether it enhances endonuclease activity, we surprisingly found that GajA activity was severely inhibited by ATP or the nonhydrolyzable analog AMP-PNP, and 1 mM ATP or AMP-PNP fully suppressed GajA activity (Figure 5A). To clarify whether the ATP inhibition was simply due to Mg^2+^ chelation, we tested the inhibitory effect of ATP in the presence of 5 mM Mg^2+^. With excess Mg^2+^, 1 mM ATP still strongly inhibited GajA cleavage, and 1.5 mM ATP fully suppressed GajA activity (Supplementary Figure S15). We further tested the effect of all sets of NTP and dNTP, including ADP and AMP, on GajA activity. The results showed that GajA activity was strongly inhibited by all NTP and dNTP, even ADP, while AMP had no significant effect (Figure 5B). Further investigation showed that other NDP also inhibited GajA endonuclease activity, while NMP, dNMP and nucleosides had no significant effect on GajA activity (Figure 5C). Apparently, the endonuclease activity of GajA is negatively regulated by nucleotides and the regulation is not dependent on the hydrolysis of ATP or other nucleotides.

### GajA functional domains

**Figure 6.**
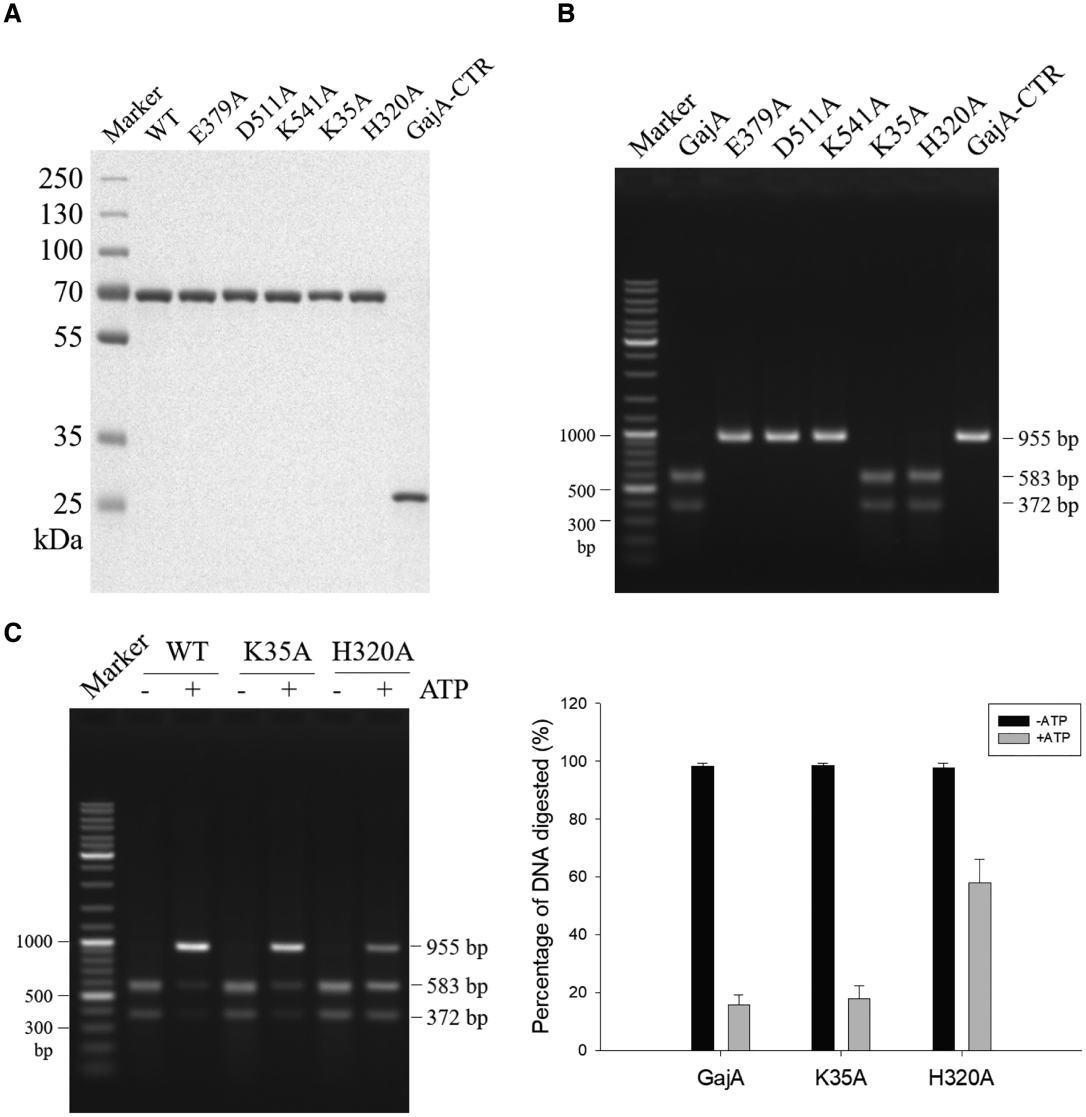
Investigation of GajA functional domains by site-specific mutagenesis. (**A**) SDS-PAGE analysis of purified wild-type (WT) GajA, GajA mutants and the C-terminal polypeptide (CTR) of GajA. (**B**) Endonuclease activity of the proteins in (A). (**C**) The effect of K35A or H320A mutations on the ATP inhibition of GajA activity. H320A but not K35A mutation partially relieved the inhibition of ATP on GajA activity. For (B) and (C), 125 ng of λ955 DNA (20 nM) was incubated with 0.2 μM GajA in a final volume of 10 μl in the optimal reaction buffer with or without 0.5 mM ATP. Reactions were performed at 37°C for 5 min and then stopped by addition of 2 μl of 6× loading dye containing 20 mM EDTA. Samples were analyzed via native agarose gel electrophoresis. Bar graphs represent the average of three independent experiments with error bars representing the standard error of the mean.

Based on sequence homology, the OLD family proteins are the closest to GajA among characterized nucleases (Supplementary Figure S16). BpOLD and TsOLD both have an ATPase and a TOPRIM domain, resembling the domain organization of GajA (23,24). As previously reported, the ATPase domain of TsOLD has ATP hydrolysis function, and the TOPRIM domain conducts non-specific nuclease activity (24). Despite the functional discrepancy between the OLD proteins and GajA, some key residues in their active sites are apparently similar. Multisequence alignment of GajA, OLD family members, and their homologs from various species suggest potential key residues in their active sites, such as the conserved TOPRIM glutamate and the DxD motif (Supplementary Figure S16). Therefore, we conducted alanine screening of conserved residues in both the ATPase-like domain and the TOPRIM domain of GajA. Single mutations K35A, H320A, E379A, D511A and K541A (sites labeled by asterisks in Supplementary Figure S17) were introduced into the wild-type GajA, respectively. In addition, we also constructed an N-terminal domain-truncated version of GajA (GajA-CTR), leaving only the TOPRIM domain (residues 348–578). GajA mutants were purified using the same procedure as that for the wild-type protein (Figure 6A). Mutations of the key residues in the TOPRIM domain (E379A, D511A and K541A) completely abolished the endonuclease activity of GajA, while those in the ATPase-like domain (K35A and H320A) showed no effect (Figure 6B), confirming that the endonuclease active site is located in the TOPRIM domain. However, the H320A mutation in the ATPase-like domain partially relieved the inhibition of ATP on GajA endonuclease activity (Figure 6C), suggesting that the ATPase-like domain mediates the regulation of endonuclease activity by nucleotide sensing. Consistently, GajA-CTR exhibited no endonuclease activity (Figure 6B), implying the indispensable role of the ATPase-like domain. Although the homologous residue in the TsOLD protein is crucial for the ATP hydrolysis (24), a mutation of K35 did not affect the inhibition by ATP (Figure 6C), supporting a model in which the binding but not hydrolysis of ATP (and other nucleotides) by the ATPase-like domain is responsible for the regulation of the endonuclease activity of GajA.

## DISCUSSION

### GajA is a novel DNA nicking enzyme

GajA exhibits specific and metal-dependent DNA cleavage activity (Figure 1D). In contrast, homologs of GajA (BpOLD, XccOLD and TsOLD) have non-specific nuclease activity (23,24). Phylogenetic analysis of GajA and its homologs from different species revealed the evolutionary relationships between GajA and BpOLD and TsOLD (Supplementary Figure S17). Although the OLD family proteins function in DNA repair and/or replication (23,24), while GajA is responsible for phage defense, they may have evolved from a common ancestor. GajA and its homologs, including OLD proteins, have conserved TOPRIM active sites, such as the conserved glutamate and the DxD motif, although the overall sequence similarity is low (Supplementary Figure S16). Like BpOLD and TsOLD with a two-metal catalysis mechanism (23,24), GajA may share a common mechanism due to the similarity of the active sites (Figure 6B).

The relationship between the Gabija and OLD family proteins is intriguing. The original work had predicted 4598 Gabija systems and GajAs therein, and the later characterized BpOLD and XccOLD were included in these potential GajAs (12). Later, work establishing the OLD family (23) also predicted 295 Class 2 OLD family proteins, of which most were included in the 4598 GajAs predicted earlier (12). However, characterized OLD family members (23) and the GajA studied in this work demonstrate clear functional divergence. At this stage, whether the GajA and OLD family proteins are actually overlapping or distinct from each other is difficult to know. There are no protein sequence features that clearly distinguish GajA and OLD family proteins from the few characterized members from each family, although in evolutionary trees they are separated (Supplementary Figure S17). More functional studies on each family are necessary to answer this question. Apparently, the sequence specificity of the characterized OLD family proteins has been diminished or lost during functional divergence. In contrast, it is likely that the ATPase-like domain of GajA has lost its ATP-hydrolysis activity but retained the nucleotide-binding function that has evolved into a regulatory domain. The binding of nucleotides by GajA seems not specific for the base and sugar ring, as all NTP and dNTP had a similar effect on GajA activity. However, the phosphate group of the nucleotide plays a crucial role in the binding and regulation, as AMP failed to inhibit GajA activity while ADP and ATP showed strong inhibition (Figure 5B). Further structural studies are required to clarify the specific mechanism of GajA function.

GajA is a natural site-specific nicking endonuclease (NEase) with the recognition sequence 5′-TNNNS↓RGGNNA-3′ (DNA single letter code, S: G/C; R: A/G; N: A/G/C/T; down arrow marks the nicking site) (Figure 4D). Known natural site-specific NEases have been divided into two major groups: one includes small HNH NEases from phage or prophage genomes that nick dsDNA sites with 3- to 5-bp specificities, for example Nt.CviPII (↓CCD) originally found in chlorella virus (34,35). These small HNH NEases are involved in phage DNA packing and pathogenicity island mobility and are widespread in nature (36,37). Other phage-encoded NEases with longer recognition sequences may also be classified into this group. This latter group includes the phage group I intron-encoded HNH homing endonucleases I-PfoP3I that nick DNA sites of 14–16 bp (38) and the T7-like phage ΦI encoded I-TslI that nick DNA sites with a 9-bp core sequence (39). The other group of NEases with 3- to 7-bp specificities are natural components of restriction systems, such as Nb.BtsI, the large subunit (B subunit) of BtsI (40). Distinct from these other natural nicking enzymes, GajA is a free-standing nicking enzyme from bacteria that relies on a TOPRIM domain to nick the DNA, and functions in bacteriophage resistance.

Type II restriction endonucleases cleave within or at short specific distances from a recognition site (7,10). They usually require magnesium for DNA cleavage. At present, all characterized Type II restriction endonucleases are classified into 12 subtypes by their function modes, namely, A (recognizes **a**symmetric sequences and cleaves within, or a defined distance away from the sequence), B (cleaves DNA on **b**oth sides of the recognition sequence, releasing a small fragment that contains the recognition sequence), C (**c**ombined enzymes containing endonuclease and methyltransferase activities in the same protein), E (Type IIP enzymes with allosteric **e**ffector domains that stimulate catalysis when bound to additional recognition sequences), F (binds two recognition sequences and cleaves coordinately, hydrolyzing all four DNA strands at once), G (with a DNA-cleavage domain and a **g**amma-class DNA-methylation domain in a single polypeptide chain), H (**h**ybrid, part Type I and part Type II), L (**l**one strand DNA modification), M (requires **m**ethylated recognition sequences), P (recognizes **p**alindromic [symmetric] DNA sequences and cleaves symmetrically within the sequence), S (cleavage is **s**hifted to one side of the sequence, within one or two turns of the double helix away), and T (acts as a he**t**erodimer, and comprises **t**wo different subunits) (7,41–43). Given the palindromic recognition sequence with an additional central base (bold) 5′-AATAACC**C**GGTTATT-3′, GajA may act similarly as a typical Type IIP restriction enzyme to cleave both strands within the recognition site and leave one-nt sticky ends on the products (Supplementary Figure S11B). However, GajA should not be considered as a restriction enzyme, since it tolerates some sequence degeneracy within its recognition sequence whereas binding and/or cleavage of restriction enzymes depend on a perfect match to the recognition sequence. The apparent restriction-enzyme-like dsDNA cleavage by GajA is due to the special arrangement of two overlapping nicking recognition sequences like that shown in Supplementary Figure S11B.

### Proposed model for the Gabija anti-phage defense mechanism

The Gabija gene cassette containing GajA and GajB genes is located between 93 871 and 97 763 of the *B. cereus* VD045 genome (AHET01000033). When this cassette is cloned into the plasmid pSG1-rfp and then transformed into the donor bacterial strain *Bacillus subtilis* str. BEST7003, the bacterium acquires a strong defense against various phages (12). Similarly, when introduced into *E. coli*, the Gabija gene cassette gave the bacteria strong resistance to bacteriophage T7 (Supplementary Figure S12A), indicating that only the two genes GajA and GajB are sufficient for bacterial defense against certain phages.

**Figure 7.**
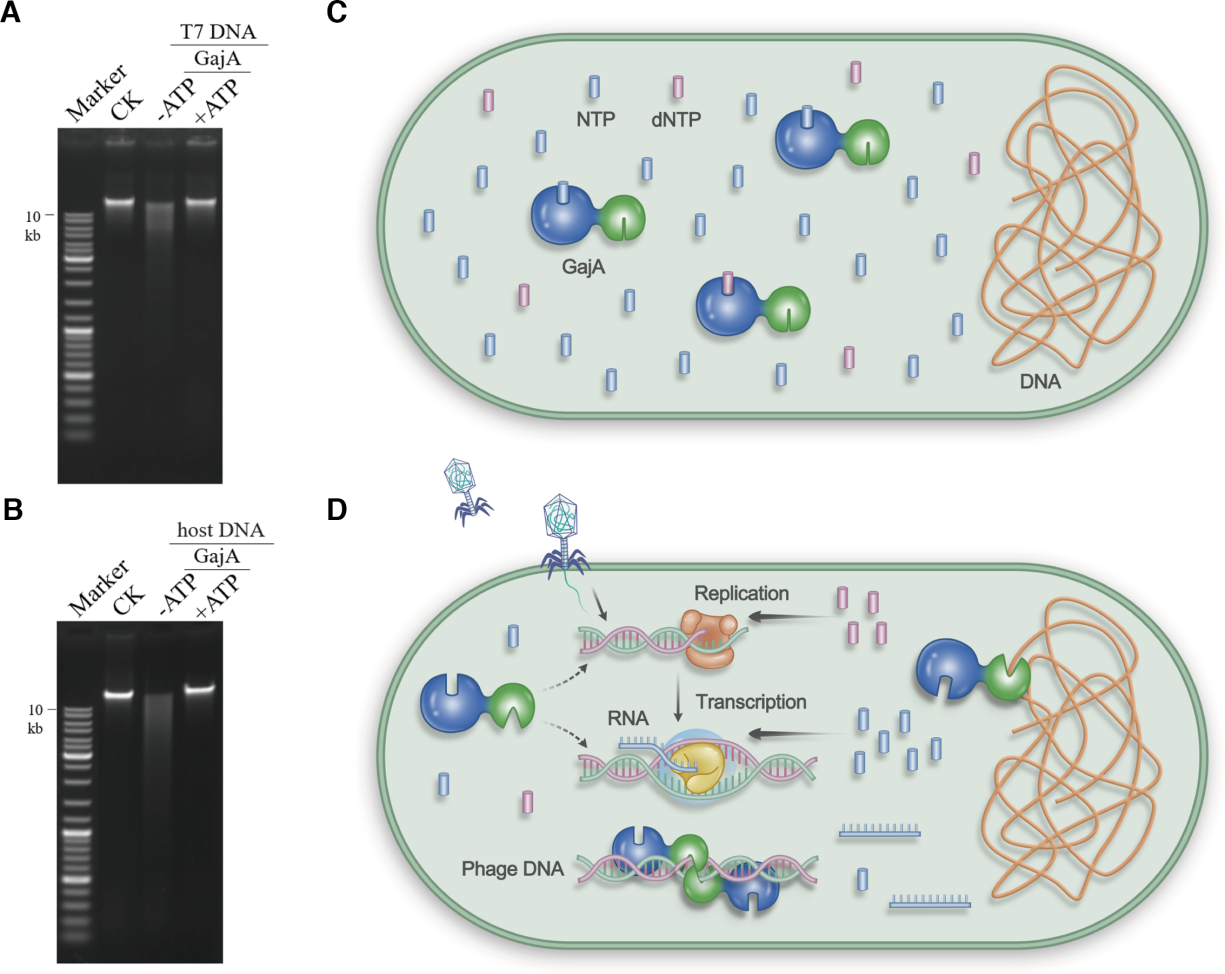
Schematic showing the proposed mechanism of Gabija anti-phage bacterial defense. The DNA nicking of GajA on the genomic DNA of bacteriophage T7 (**A**) or *E. coli* B (ATCC^®^ 11303™) (**B**) in the absence or presence of 0.5 mM ATP. (**C**) Under normal conditions, GajA endonuclease activity is fully inhibited by nucleotides at physiological concentrations in bacteria. The ATPase-like domain of GajA senses and binds NTP and dNTP to allosterically regulate the TOPRIM domain. (**D**) During phage invasion, active phage transcription and DNA replication deplete cellular NTP and dNTP. When NTP and dNTP concentrations decrease to a certain degree, the loss of nucleotide binding of the GajA ATPase-like domain activates the TOPRIM domain. The latter, in turn, mediates phage DNA cleavage and may also mediate destruction of bacterial genomic DNA for abortive infection. GajB may contribute to GajA activation or facilitate GajA cleavage, which is under investigation and not shown in this model.

In the present study, we characterized the function of GajA. GajA is a site-specific nicking enzyme and is negatively regulated by nucleotide concentrations. GajA efficiently catalyzes DNA nicking on both T7 and *E. coli* genomic DNA *in vitro*, as judged from the smear of native gel bands of the GajA-treated genomic DNA (Figure 7A and B). However, in the presence of only 0.5 mM ATP, such nicking activity was inhibited (Figure 7A and B). The physiological concentration of ATP is over 3 mM and the total nucleotide concentration is above 8.7 mM in *E.coli* at mid-log phase (44), while GajA activity is fully inhibited by 1 mM ATP *in vitro*. Therefore, the robust DNA nicking activity of GajA should be strictly suppressed by NTP and dNTP at physiological concentrations. Indeed, overexpression of GajA or even its H320A mutant, which partially relieves the nucleotide inhibition, results in no toxicity to *E. coli*, as no difference in the bacterial growth curve was observed with/without GajA overexpression, indicating that the GajA activity is tightly suppressed *in vivo*. It may be that only drastic changes in cellular nucleotide concentrations can activate the GajA endonuclease activity. Phages often supply their own nucleases to degrade host nucleic acids to supply the building blocks for their own genomes. In this scenario, the cellular concentrations of the degradation products, NMP and dNMP, might be temporarily high. Interestingly, GajA can avoid inhibition from such nucleoside monophosphates (Figure 5B).

Our data on GajA function suggest a model for part of the anti-phage mechanism of the Gabija system (Figure 7C and D). GajA endonuclease activity is fully inhibited by nucleotides in the physiological state (Figure 7C). The robust transcription and DNA replication of invading phages deplete the cellular NTP and dNTP, releasing the allosteric suppression and activating the GajA endonuclease activity, and in turn resulting in the cleavage of phage DNA (Figure 7D). Consistently, GajA recognition sequences have been identified in phages previously reported as targets of the Gabija system (Supplementary Table S7). Meanwhile, GajA recognition sequences also appear in host bacterial genomes, indicating that GajA may also destruct genomic DNA of bacteria for abortive infection. The Gabija system, which relies on a nucleotide-sensing endonuclease, represents a concise strategy for anti-phage defense.

The other component of the Gabija bacterial defense system, GajB, is predicted to be a UvrD-like helicase whose function remains to be understood. The predicted GajA and GajB genes are separated by one nucleotide, and when the whole Gabija gene cassette in its native formation was overexpressed in *E. coli*, we observed only GajA but not GajB expression. The individual GajB gene can be expressed in *E. coli* and the GajB protein purified, but at this stage in our assays no helicase activity has yet been detected. Adding GajB into GajA reactions also did not show any effect on DNA nicking or nucleotide inhibition. However, resistance to bacteriophage T7 by *E. coli* requires the whole Gabija gene cassette, indicating that GajB is also necessary. Although in our model GajA seems sufficient for bacteriophage resistance, in an actual bacterium, reducing the cellular nucleotide concentration from several mM to under 1 mM is too drastic, even after phage invasion. And considering the molecular crowding in cells and the sensitivity of GajA to salt (Supplementary Figure S5), activating GajA in time to resist a virulent phage like T7 is difficult and is likely dependent on GajB. It is reasonable to speculate that GajB as a helicase may interact with GajA and somehow stimulate/facilitate the binding, cleavage, and/or turnover of GajA on its recognition sites during DNA translocation of GajB (inefficient turnover of GajA supports this hypothesis); GajB may also separate annealed DNA strands after DNA nicking by GajA. These possibilities are currently under investigation to determine the complete Gabija bacterial defense mechanism.

## Supplementary Material

gkab277_Supplemental_File
