# Supplementary material for: A nucleotide-sensing endonuclease from the Gabija bacterial defense system
Source: Nucleic Acids Res. 2021 Apr 22;49(9):5216–29. doi: 10.1093/nar/gkab277 (PMC8136825; doi:10.1093/nar/gkab277)
Supplement: gkab277_Supplemental_File [file gkab277_supplemental_file.pdf]

Supplementary Materials

A nucleotide-sensing endonuclease from the Gabija bacterial defense system

Rui Cheng, Fengtao Huang, Hui Wu, Xuelin Lu, Yan Yan, Bingbing Yu, Xionglue Wang and Bin Zhu*

Key Laboratory of Molecular Biophysics, the Ministry of Education, College of Life Science and Technology and Shenzhen College, Huazhong University of Science and Technology, Wuhan, Hubei 430074, China

*To whom correspondence should be addressed. Email: bin_zhu@hust.edu.cn

Table S1. Sequence (5′-3′) of the GajA gene from *B. cereus* VD045.

B. cereus VD045 GajA gene sequence (located between 94190 and 95926 of the *B. cereus* VD045 genome [AHET01000033])
ATGAAATTCAGTAATATTACAATAAAGAACTTCAGGAATTTTGAAAAAGTAAATATAAATT<br>TAGATAATAAAAATGTGATTTTCGGGATGAATGATATTGGAAAAACAAATTTTTTATATGC<br>ATTGAGATTTCTTTTAGATAAAGAGATAAGAAAATTCGGTTTTAATAAATCTGATTATCATA<br>AACATGACACTTCTAAAAAAATTGAAATTATTTTAACACTTGATTTGTCTAATTATGAAAA<br>GGATGAAGATACAAAAAAACTTATTTCAGTGGTTAAGGGTGCTAGAACATCGGCAAATGC<br>AGATGTTTTTTATATCGCACTAGAATCTAAATATGATGATAAAGAATTATATGGGAACATA<br>ATTTTAAAATGGGGATCGGAACTAGATAATTTAATAGATATACCAGGGAGAGGGAACATA<br>AACGCGTTAGATAATGTATTTAAGGTGATTTATATAAATCCGCTTGTTGATTTAGACAAATT<br>GTTCGCACAAAATAAAAAATATATTTTTGAAGAGTCACAGGGTAATGAATCAGATGAAGG<br>GATTTTAAATAATATTAAATCTTTAACAGATCAAGTAAATCAACAAATAGGAGAAATGACA<br>ATTATTAAGGGTTTCCAGCAAGAGATAACAAGTGAATATAGGTCTTTAAAAAAAGAAGAG<br>GTTTCTATTGAGCTGAAGTCCGAAATGGCAATTAAAGGATTTTTCTCAGATATTATTCCATA<br>TATAAAAAAAGACGGTGATTCTAATTACTATCCAACCTCAGGGGATGGTAGAAGAAAAAT<br>GCTTTCTTACTCTATATATAACTATCTGGCTAAGAAAAAATATGAGGATAAAATTGTTATTT<br>ATTTAATTGAGGAACCCGAAATTAGTCTACATAGATCAATGCAAATTGCTTTATCAAAACA<br>GTTATTTGAACAATCTACATATAAATATTTTTTCTTATCCACTCACTCTCCTGAACTTCTTTA<br>TGAAATGGATAATACAAGATTAATAAGAGTGCATTCAACTGAAAAGGTTGTATGTTCTTCC<br>CATATGTATAATGTGGAAGAAGCCTATGGAAGTGTCAAGAAAAAGCTAAATAAAGCTTTA<br>TCATCGGCTCTATTTGCTGAAAGAGTACTTTTAATAGAAGGTCCTTCAGAAAAAATATTATT<br>TGAAAAGGTTTTAGACGAAGTAGAACCAGAATATGAATTAAATGGAGGTTTCTTGCTTGAA<br>GTAGGAGGGACGTACTTTAATCATTATGTGTGTACATTAAATGATTTAGGTATAACCCATA<br>TAATTAAAACAGATAATGATTTGAAATCAAAAAAAGGTAAAAAAGGTGTATACGAATTAC<br>TAGGATTAAATAGATGCTTAAACTTATTAGGACGTGAAAATCTAGATGAGATTACTATTGA<br>CATCCCTGAAGATATAAAAGGTAAGAAGAAAAAAGAGAGACTTAATGAAAGAAAAAAAG<br>AGATTTTTAAACAATATAAAAATGAGGTAGGGGAATTCTTAGGGGAACGAATATATTTATC<br>GGAAATCGATCTGGAAAATGATTTATATTCTGCAATTGGTGAAAGCATGAAAAGAATTTTT<br>GAAAACGAAGATCCCGTGCACTATTTACAGAAAAGTAAACTATTTAACATGGTCGAGCTAG<br>TAAATAATTTAAGTACTAAAGATTGTTTTGATGTTTTTGAGCACGAAAAATTTGCATGCCTA<br>AAGGAGTTGGTGGGTAGTGATAGAGGATGA

Table S2. Primers for construction of GajA and its mutants.

**Name**	**Sequence**
GajAgene-F	GCCATATGAAATTCAGTAATATTACAATAAAGAAC
GajAgene-R	GGCCGCTCATCCTCTATCACTACCCACCAACTCC
GajAplasmid-F	AGAGGATGAGCGGCCGCACTCGAGCACCACC
GajAplasmid-R	GAATTTCATATGGCTGCCGCGCGGCAC
GajA-K35A-F	ATTGGAGCAACAAATTTTTTATATGCATTGAG
GajA-K35A-R	ATTTGTTGCTCCAATATCATTCATCCCGAAAATC
GajA-H320A-F	ATCCACTGCCTCTCCTGAACTTCTTTATG
GajA-H320A-R	GGAGAGGCAGTGGATAAGAAAAAATATTTATATG
GajA-E379A-F	TTAATAGCAGGTCCTTCAGAAAAAATATTATTTG
GajA-E379A-R	AGGACCTGCTATTAAAAGTACTCTTTCAGCAAATAG
GajA-D511A-F	TATTTATCGGAAATCGCTCTGGAAAATG
GajA-D511A-R	GATTTCCGATAAATATATTCGTTC
GajA-K541A-F	TATTTACAGAAAAGTGCACTATTTAACATGG
GajA-K541A-R	ACTTTTCTGTAAATAGTGCACGGG
GajA-CTR-F	TATACCATGTATAATGTGGAAGAAGCCTATGGAAG
GajA-CTR-R	ATTATACATGGTATATCTCCTTCTTAAAGTTAAAC

Table S3. Primers for PCR amplification of DNA substrates from λDNA and pUC19 plasmid.

**Name**	**Sequence**
λ1-F	CGACCTCGCGGGTTTTCG
λ1-R	GTGGTGCTGGTCTGGTCAG
λ2-F	GGACACCTCCAGCCGTAAG
λ2-R	GAGCACTGTCTTCCTGACG
λ3-F	GATTACAACACGCTGATGGC
λ3-R	CCACGAATGCCGATTTGCC
λ4-F	CCAGCACGCGTTATCTTGG
λ4-R	AAAGGACGGTTATCACATTC
λ955-F	ATCCAAGCTTATGGGCCGCCACGAC
λ955-R	CTAGACCCTCCAAATCCGCTGCCAC
pUC19-F	ACCCCCCGTTCAGCCCGACCGCTG
pUC19-R	CGGAGGACCGAAGGAGCTAACCGC

Table S4. Sequences (5′-3′) of sox7 RNA.

The sox7 RNA sequence (GenBank: NM_031439.4)
GGGAGACCCUCGAGGACAGAUCGCCUGGAGACGGCAAGAGCCGCCACCAUGAAAAGGCC<br>GGCGGCCACGAAAAAGGCCGGCCAGGCAAAAAAGAAAAAGGGUUCUGGAGCUUCGCUGC<br>UGGGAGCCUACCCUUGGCCCGAGGGUCUCGAGUGCCCGGCCCUGGACGCCGAGCUGUCG<br>GAUGGACAAUCGCCGCCGGCCGUCCCCCGGCCCCCGGGGGACAAGGGCUCCGAGAGCCG<br>UAUCCGGCGGCCCAUGAACGCCUUCAUGGUUUGGGCCAAGGACGAGAGGAAACGGCUGG<br>CAGUGCAGAACCCGGACCUGCACAACGCCGAGCUCAGCAAGAUGCUGGGAAAGUCGUGG<br>AAGGCGCUGACGCUGUCCCAGAAGAGGCCGUACGUGGACGAGGCGGAGCGGCUGCGCCU<br>GCAGCACAUGCAGGACUACCCCAACUACAAGUACCGGCCGCGCAGGAAGAAGCAGGCCA<br>AGCGGCUGUGCAAGCGCGUGGACCCGGGCUUCCUUCUGAGCUCCCUCUCCCGGGACCAG<br>AACGCCCUGCCGGAGAAGAGAAGCGGCAGCCGGGGGGCGCUGGGGGAGAAGGAGGACAG<br>GGGUGAGUACUCCCCCGGCACUGCCCUGCCCAGCCUCCGGGGCUGCUACCACGAGGGGC<br>CGGCUGGUGGUGGCGGCGGCGGCACCCCGAGCAGUGUGGACACGUACCCGUACGGGCUG<br>CCCACACCUCCUGAAAUGUCUCCCCUGGACGUGCUGGAGCCGGAGCAGACCUUCUUCUC<br>CUCCCCCUGCCAGGAGGAGCAUGGCCAUCCCCGCCGCAUCCCCCACCUGCCAGGGCACCC<br>GUACUCACCGGAGUACGCCCCAAGCCCUCUCCACUGUAGCCACCCCCUGGGCUCCCUGGC<br>CCUUGGCCAGUCCCCCGGCGUCUCCAUGAUGUCCCCUGUACCCGGCUGUCCCCCAUCUCC<br>UGCCUAUUACUCCCCGGCCACCUACCACCCACUCCACUCCAACCUCCAAGCCCACCUGGG<br>CCAGCUUUCCCCGCCUCCUGAGCACCCUGGCUUCGACGCCCUGGAUCAACUGAGCCAGG<br>UGGAACUCCUGGGGGACAUGGAUCGCAAUGAAUUCGACCAGUAUUUGAACACUCCUGGC<br>CACCCAGACUCCGCCACAGGGGCCAUGGCCCUCAGUGGGCAUGUUCCGGUCUCCCAGGU<br>GACACCAACGGGUCCCACAGAGACCAGCCUCAUCUCCGUCCUGGCUGAUGCCACGGCCA<br>CGUACUACAACAGCUACAGUGUGUCAGGAUCCCCCAAGAAGAAGAGGAAAGUCUCGAGC<br>GACUACAAAGACCAUGACGGUGAUUAUAAAGAUCAUGACAUCGAUUACAAGGAUGACG<br>AUGACAAGGCUGCAGGAUGAA

Table S5. Sequences (5′-3′) of the substrates used in Figure S7 (all substrates contain a recognition sequence of GajA [underlined]).

dsDNA sequence
TTTTTTTTTTTTTTTTTAATAACCCGGTTATTTTTTTTTTTTTTTTTTTTTTTT
ssDNA-F sequence
TTTTTTTTTTTTTTTTTAATAACCCGGTTATTTTTTTTTTTTTTTTTTTTTTTT
ssDNA-R sequence
AAAAAAAAAAAAAAAAAAAAAAAAAATAACCGGGTTATTAAAAAAAAAAAAAAAAA
dsRNA sequence
GGGAAACCCUCCGUAAGUAUCCUCCUUUAAUUGCUUUAUUCAGAAAAGCAUCACAAACA UACCGUUUACCUGACAACCUAAUACUGGAAAAAGGCCAAAAAAUAGUAAUUCCAAUUU ACUCACUCCAUUUCGAUGAUAAAUAUUUCGAGGAUCCUCAAAAAUUCGAUCCUGAAAGA UUUUCACCCGAAAACAAAGAUAAACGUCCUAAUGGUGUUUAUCUUCCAUUUGGUGAUG GACCUAGAAUGUGUAUAGGAAAACGUUUUGCUGAGAUGGAAAUGAGAUUGGCUUUACU CGAAAUGUUGAGCAAAUUUGAAGUCCUACCAUGUGAAAAAACAGAAGUUCCUCUAAAA UAUUCUAACAAAGUUUUAACAUUGAUGAAUAACCCGGUUAUUCCAAAACAUGGAAUUU GGUUAAAAUUUCAAAAAAUUGCUUAACUUUAGUUAAGAAUUAUGUAUUUGAACAAAAA AAAAUUAACAGUUCGUAACCUGAGAAAAUUGUGUGGAUUUUACUAUAUAUGAUAUAUA UUAUAUUUGUAUAAUUUCAAAAAUAUUAUAAUAAUAUGUAUUGAAUUUGUAAGUAUAC AAUCUUUAUUUUAAAUAC
(+) strand RNA sequence
GGGAAACCCUCCGUAAGUAUCCUCCUUUAAUUGCUUUAUUCAGAAAAGCAUCACAAACA UACCGUUUACCUGACAACCUAAUACUGGAAAAAGGCCAAAAAAUAGUAAUUCCAAUUU ACUCACUCCAUUUCGAUGAUAAAUAUUUCGAGGAUCCUCAAAAAUUCGAUCCUGAAAGA UUUUCACCCGAAAACAAAGAUAAACGUCCUAAUGGUGUUUAUCUUCCAUUUGGUGAUG GACCUAGAAUGUGUAUAGGAAAACGUUUUGCUGAGAUGGAAAUGAGAUUGGCUUUACU CGAAAUGUUGAGCAAAUUUGAAGUCCUACCAUGUGAAAAAACAGAAGUUCCUCUAAAA UAUUCUAACAAAGUUUUAACAUUGAUGAAUAACCCGGUUAUUCCAAAACAUGGAAUUU GGUUAAAAUUUCAAAAAAUUGCUUAACUUUAGUUAAGAAUUAUGUAUUUGAACAAAAA AAAAUUAACAGUUCGUAACCUGAGAAAAUUGUGUGGAUUUUACUAUAUAUGAUAUAUA UUAUAUUUGUAUAAUUUCAAAAAUAUUAUAAUAAUAUGUAUUGAAUUUGUAAGUAUAC AAUCUUUAUUUUAAAUAC
(-) strand RNA sequence
GUAUUUAAAAUAAAGAUUGUAUACUUACAAAUUCAAUACAUAUUAUUAUAAUAUUUUU GAAAUUAUACAAAUAUAAUAUAUAUCAUAUAUAGUAAAAUCCACACAAUUUUCUCAGG UUACGAACUGUUAAUUUUUUUUUGUUCAAAUACAUAAUUCUUAACUAAAGUUAAGCAA UUUUUUGAAAUUUUAACCAAAUUCCAUGUUUUGGAAUAACCGGGUUAUUCAUCAAUGU UAAAACUUUGUUAGAAUAUUUUAGAGGAACUUCUGUUUUUUCACAUGGUAGGACUUCA AAUUUGCUCAACAUUUCGAGUAAAGCCAAUCUCAUUUCCAUCUCAGCAAAACGUUUUCC UAUACACAUUCUAGGUCCAUCACCAAAUGGAAGAUAAACACCAUUAGGACGUUUAUCUU UGUUUUCGGGUGAAAAUCUUUCAGGAUCGAAUUUUUGAGGAUCCUCGAAAUAUUUAUC AUCGAAAUGGAGUGAGUAAAUUGGAAUUACUAUUUUUUGGCCUUUUUCCAGUAUUAGG UUGUCAGGUAAACGGUAUGUUUGUGAUGCUUUUCUGAAUAAAGCAAUUAAAGGAGGAU ACUUACGGAGGGUUUCCC
DNA sequence used as the transcription template of the (+) strand RNA
TTAATTGGGCCACCTATAGGGAAACCCTCCGTAAGTATCCTCCTTTAATTGCTTTATTCAGA AAAGCATCACAAACATACCGTTTACCTGACAACCTAATACTGGAAAAAGGCCAAAAAATA GTAATTCCAATTTACTCACTCCATTTCGATGATAAATATTTCGAGGATCCTCAAAAATTCGA TCCTGAAAGATTTTCACCCGAAAACAAAGATAAACGTCCTAATGGTGTTTATCTTCCATTTG GTGATGGACCTAGAATGTGTATAGGAAAACGTTTTGCTGAGATGGAAATGAGATTGGCTTT ACTCGAAATGTTGAGCAAATTTGAAGTCCTACCATGTGAAAAAACAGAAGTTCCTCTAAAA TATTCTAACAAAGTTTTAACATTGATGAATAACCCGGTTATTCCAAAACATGGAATTTGGTT AAAATTTCAAAAAATTGCTTAACTTTAGTTAAGAATTATGTATTTGAACAAAAAAAAATTA ACAGTTCGTAACCTGAGAAAATTGTGTGGATTTTACTATATATGATATATATTATATTTGTA TAATTTCAAAAATATTATAATAATATGTATTGAATTTGTAAGTATACAATCTTTATTTTAAA TAC

Table S6. The 110 nicking sites in T7 genomic DNA by GajA revealed by run-off sequencing (down arrows mark the nicking site).

ATGATACC↓GGGTCGGT	GCTGGATG↓GGGTGATA	ACGTTACC↓AGTGACAA
ATTTTGGC↓GGTGTAAG	GGTTACTC↓AGGGATTG	ACATTGAG↓GGCTTAGT
ATTTCACC↓AGTGTTCT	CGATATAC↓AGGGCTAC	AATTACAC↓GGGGTGAA
AGATTACC↓ACTATATA	AAATTCCG↓GGTGTACG	TATGATGC↓AGGTTAAC
CTTTAAAC↓GGGTTAGT	AGATGAAG↓GGTGAATA	TTTCAACG↓GGGACATT
CAGTATAG↓GGACAATG	CCACTGCG↓GGGTTATC	GCACAACC↓GGGACTCT
TTATATAG↓TGGTAATC	AAATTCCC↓GGCTGTAA	CTATCATG↓GGGTCTTC
CTATAGAG↓GGACAAAC	CGATACCG↓AGGGTCAC	TGATTGCC↓GCTCAGAC
CGTTTGAC↓GGGTCTTG	ACTTCACC↓GCCAAATA	AATACGTC↓CCACATTC
GTATTACC↓AGTTAACT	GATTTACC↓GCTGGAGA	TAGGGACC↓GGACAATA
GTCACCAC↓GGGTGATA	TTATTGGC↓AGGGAAGA	ACTTGGAG↓GGACGCAT
TTTTTCCC↓AGGCTTCA	AATTAGTC↓GTCACTGT	ACTCAAAC↓GGGATATT
GTACTGGC↓GGGTTTTC	GGATTAGC↓GGTCTTGA	ATTGCACC↓AGCCAATC
TTATTTGC↓GGGGCAGA	AACTTTCC↓GCTCAGCG	ATGTTACC↓GCCTATCG
GAATAGTC↓AGGTCAAC	TCCTAAGC↓GGTTACGG	CGCTGTCC↓GGGTCTTG
ATGGAACC↓AGAGAATA	TTCGGGAG↓GGGCAAAA	TGATTACC↓GAGTCACG
GTATCACC↓AGTAAAGC	ACATTAAC↓GGTAAAGA	ACGTATGC↓AGGTCATC
GAACTAGG↓AGGGAATT	AAATAAGG↓AGGCTCTA	ACCACTAG↓GGTCAATG
ACACAAGG↓GGTATAAA	AGATTGCC↓GGTCTGTG	GTGAATAC↓GGGTCATC
GGATAGAC↓GGATAGTT	CTTTAAGG↓AGACAATA	AAATCACC↓ACTCAATG
CGTTTTCC↓GGGCAACT	TTACAGCC↓GGGAATTT	CCATAGGG↓GGTACCTA
TTAATGCG↓AGGGTATT	CTGTAAGC↓GCCCAATG	CCCCATAG↓GGGGTACC
TCTTTACC↓AGCACCAA	CCAACCAC↓AGGGAATC	GCATTACC↓AGTGGCTT
CAATCGCG↓GGGGTGTT	GTTCAGAG↓GGGTAGTC	AAGTAAGG↓AGGCAATG
ATGTTACC↓AGTGGTAC	TAAATACC↓GGAACTTC	AAGTGGGG↓AGGTAAAT
TTATGAAC↓GGGTTATT	CACCACAG↓GGAGAATA	CTTTAAGG↓AGGTCAAA
GTCTCCAC↓AGGTAAAT	GGTGAGCC↓GGAGTGAA	CTCTAGAG↓AGGTAATG
GCTTCACC↓AGTACCAC	TGATAGCC↓AGTGGGTC	GGACCCAC↓GGGCTATG
GGAGCTAC↓GGGCTACA	TCCTTAGC↓GGTATACA	TTATAATG↓GGCATCAT
AGGTAGAC↓AGGCTAAA	TGGCATAC↓GGGCAATC	CGATTTGC↓AGCGTATA
TAATTCCG↓AGGACATG	TCATCACC↓AGTGAACG	ACATCTAC↓GGGATTGT
TAATCAGG↓AGGTTATC	CCAATTCC↓AGCCAATC	AAATCCAC↓GGTGCAAA
CTTTAAGG↓AGGTATAA	ATAATACC↓AGCACTCA	ACTTTAGG↓AGGATACT
GCATTTGG↓AGGTAAGA	TTCTTCAC↓GGGACATC	ACGTTAGG↓AGGTGACT
GGATACCG↓AGGGCACC	CCATGAGC↓GGGCAATC	GTCCAACG↓GGGCAACC
GAGATAGG↓GGCCTTTA	GTTTTACC↓AGTAGACC	TCGACACC↓GGGGTCAA
GAATAACC↓CGTTCATA	GCTTTGAG↓AGCCAAAA	

Table S7. Predicted GajA recognition sequences in the genomes of related phages and bacteria.

**organisms**	**The recognition sequences of GajA (underlined) contained in genomes**
Bacteriophage phi29	CATAAATCCGGGATTATGG<br>TATTAACCGGGGC
Bacteriophage SpBeta	GCATTAACCAGGTATTTTATG<br>AGTATACCAGGATTTCC<br>CGTTTACCCGGAAAAATT<br>TGAAACCAGGTATTTTA<br>CGAAACCAGGAATACT
Bacteriophage phi105	TATAATACCGGGTTTTTCA<br>AATTAAACCAGGAGA<br>ACAACCAGGTAAATCT<br>TGTCCCGGATTTACC
Bacteriophage SBSphiC	CCATTTCCCGGATTTTAT<br>TCTTTAACCGGGTATCA<br>TGTTTTACCGGGTTTGG<br>CTTTAAACCCGGAAGT<br>CCATTACCCGGATAGT<br>CCTTTTCCCGGAATCC<br>CGTTATCCGGGTTTGA
Bacillus subtilis BEST7003	TTTTTTACCCGGAATATCG<br>ATTTAATCCCGGATTATCA<br>AGAAATTCCCGGAATCG<br>AGAATACCCGGATATTAA<br>CCAATACCCGGATAAGC<br>AGAAATCCCGGATAAATA<br>GGAATACCCGGAATAGC<br>ATCTATCCCGGTAATGG<br>TCATACCCGGTAAATC<br>TCTTTCCCGGTTTATCAC
Bacillus cereus VD045	AAATAAACCCGGTAATTCA<br>TAAAATACCTGGTTGC<br>GAATACCTGGAACA<br>CCATACCAGGTTTTG<br>TGTTCCTGGTAATACA

Table S8. Predicted GajA recognition sequences in the bacteriophage λ genome.

**organisms**	**The recognition sequences of GajA (underlined) contained in bacteriophage λ**
Bacteriophage λ	CGAATAACCCGGATATTTT<br>AATTTTACCTGGTTATCG<br>TGTATTCCCGGATTAACT<br>GGTTACCTGGATTTTTT<br>GCTTTCCCGGAATTACG<br>CCTACCCGGATATTAT<br>AGTCCCGGATTTATC<br>TGTCCGGGAATAATT

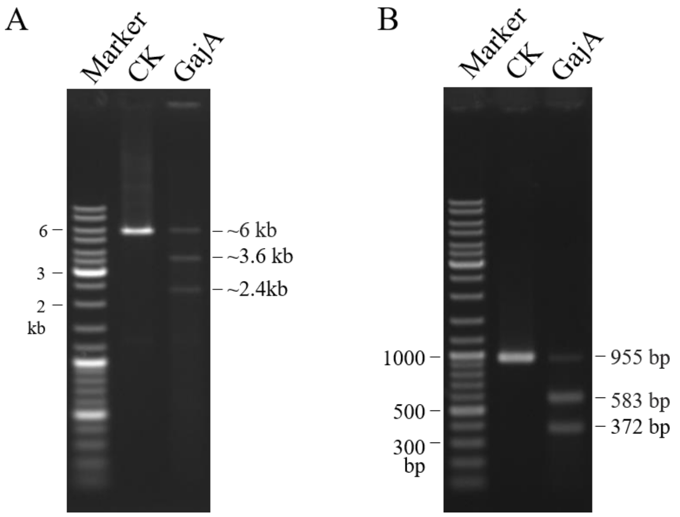

Figure S1. Specific cleavage activity of the GajA protein on λDNA. (**A**) An approximately 6 kb fragment from λDNA, named λ1, was cleaved into two specific fragments when treated by GajA. (**B**) A 955 bp fragment from λDNA, called λ955, was cut into two specific fragments by GajA. 125 ng DNA was incubated with 0.2 μM GajA in a final volume of 10 μl. Reactions were performed at 37 ℃ for 5 min and then stopped by the addition of 2 μl of 6× loading dye containing 20 mM EDTA. Samples were analyzed via native agarose gel electrophoresis.

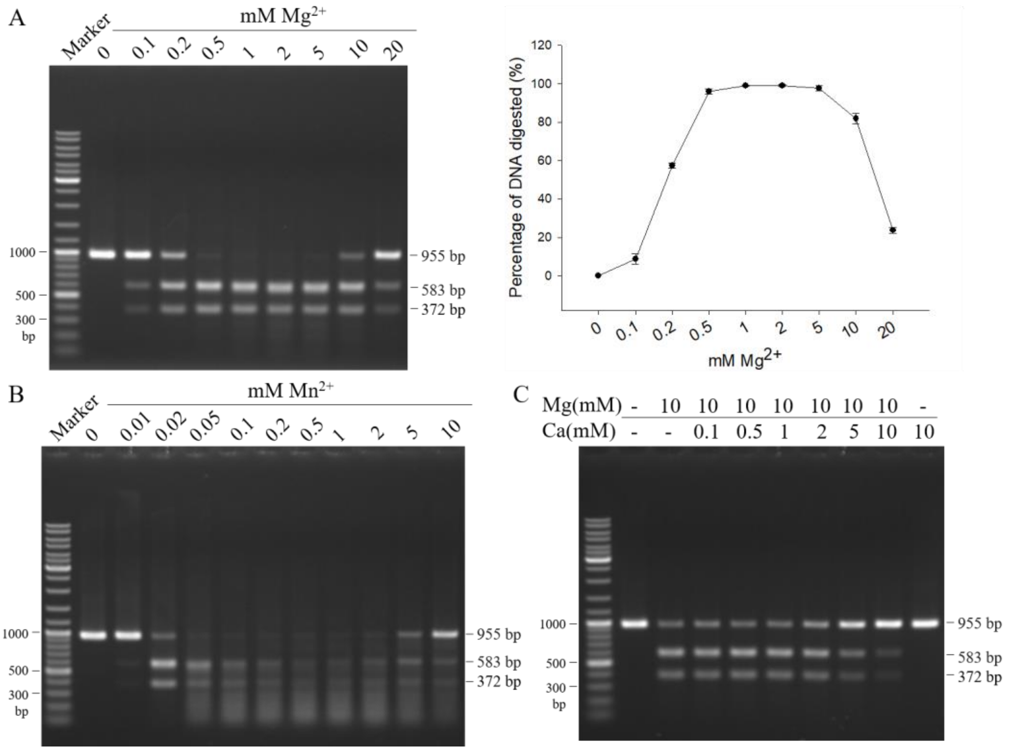

Figure S2. Nuclease activity of GajA protein in the presence of magnesium, manganese and calcium ions. (**A**) Optimized Mg^{2+} concentration for GajA endonuclease activity. DNA substrates cleaved at various Mg^{2+} concentrations were quantified and shown below. (**B**) GajA specific cleavage activity was only observed at 10-20 μM Mn^{2+}, while non-specific cleavage occurred in the presence of a wide range of Mn^{2+} concentrations. (**C**) Calcium ions compete with magnesium ions to inhibit the endonuclease activity of GajA. For A–C, 125 ng of λ955 DNA (20 nM) was incubated with 0.2 μM GajA in a final volume of 10 μl. Reactions were performed at 37 ℃ for 5 min (for A and B) or 1 min (for C), and then stopped by the addition of 2 μl of 6× loading dye containing 20 mM EDTA. Samples were analyzed via native agarose gel electrophoresis.

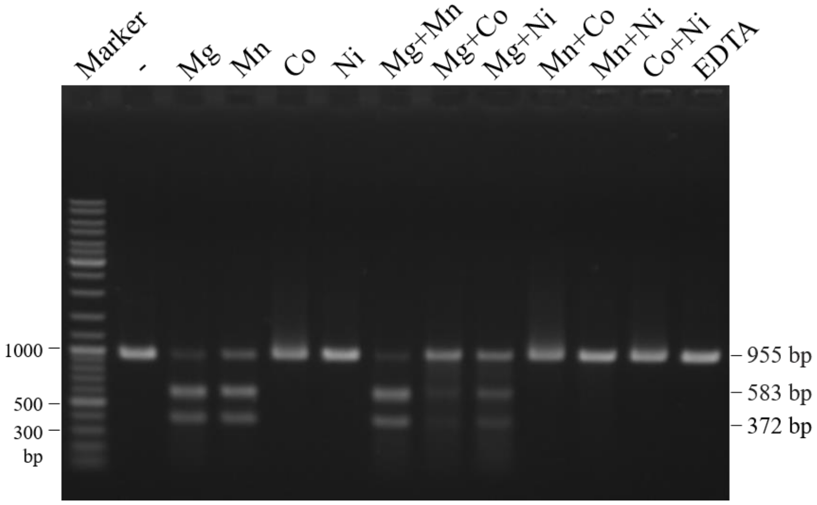

Figure S3. Nuclease activity of GajA using physiologically-relevant divalent ion concentrations. Ion concentrations: Mg^{2+}: 5 mM, Mn^{2+}: 15 μM, Co^{2+}: 0.5 mM, Ni^{2+}: 0.5 mM, respectively. 125 ng of λ955 DNA (20 nM) was incubated with 0.1 μM GajA in a final volume of 10 μl. Reactions were performed at 37 ℃ for 5 min and then stopped by the addition of 2 μl of 6× loading dye containing 20 mM EDTA. Samples were analyzed via native agarose gel electrophoresis.

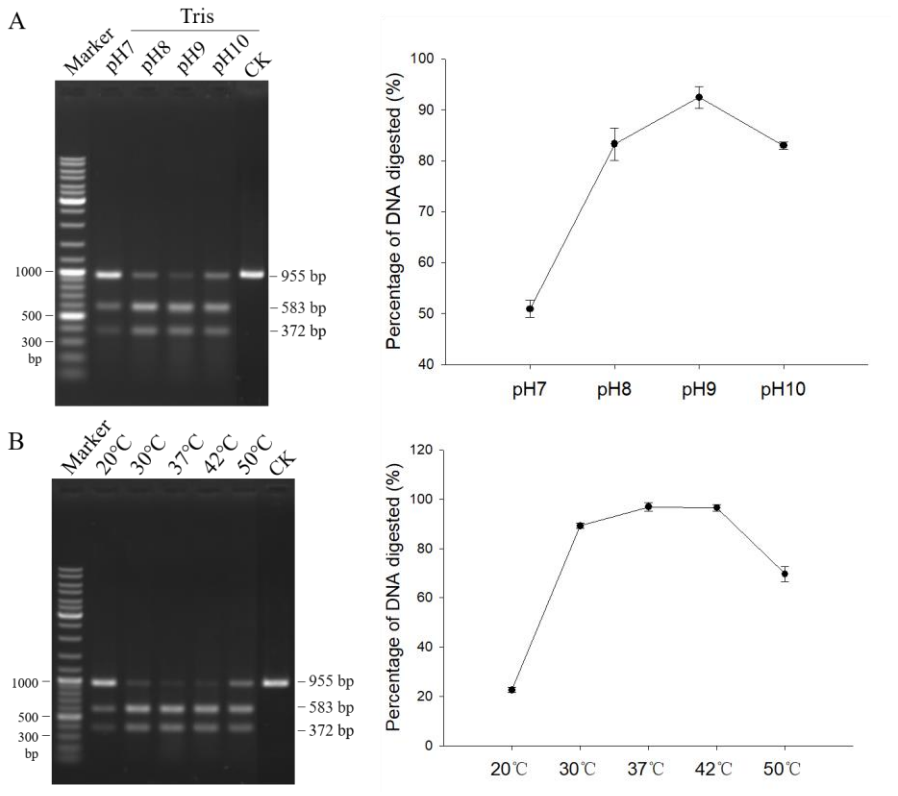

Figure S4. Optimal pH and temperature for GajA endonuclease activity. (**A**) GajA exhibits its highest activity at pH 9, and (**B**) at 37 ℃ and 42 ℃. CK refers to the control reaction without GajA at pH 9 and 37℃. In these reactions, 125 ng of λ955 DNA (20 nM) was incubated with 0.2 μM GajA in a final volume of 10 μl. Reactions were performed at 37 ℃ for 3 min and then stopped by the addition of 2 μl of 6× loading dye containing 20 mM EDTA. Samples were analyzed via native agarose gel electrophoresis.

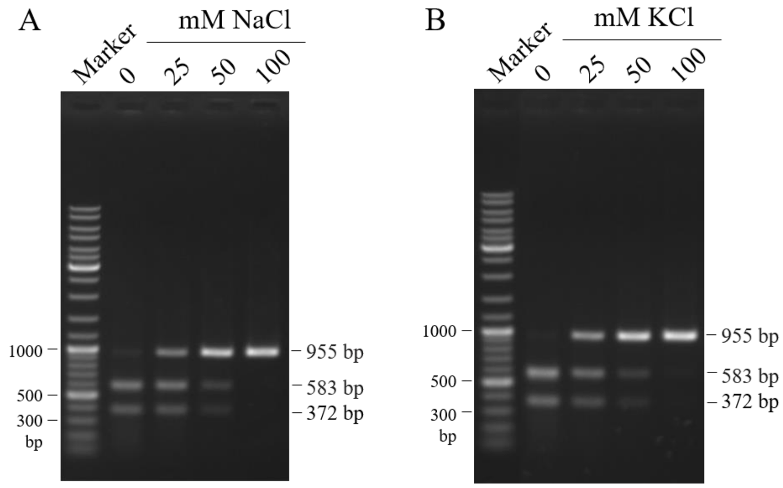

Figure S5. Effect of salts on GajA activity. GajA activity was inhibited by NaCl (**A**) or KCl (**B**). λ955 DNA was used as the substrate. 125 ng of λ955 DNA (20 nM) was used as the substrate and incubated with 0.2 μM GajA in a final volume of 10 μl in the optimal reaction buffer. Reactions were performed at 37 ℃ for 5 min and then stopped by the addition of 2 μl of 6× loading dye containing 20 mM EDTA. Samples were analyzed via native agarose gel electrophoresis.

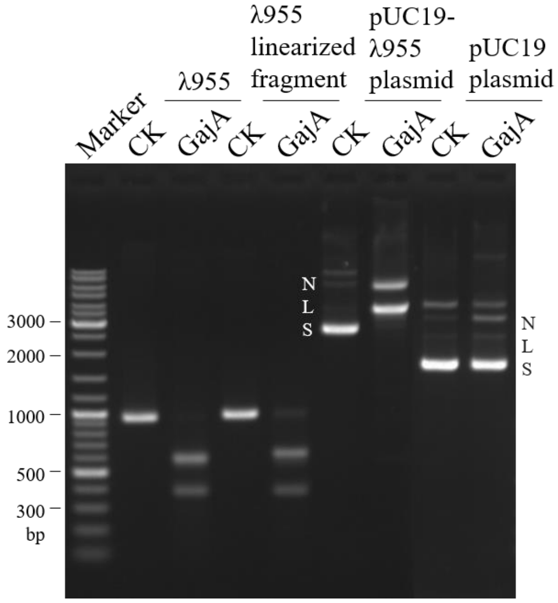

Figure S6. Nuclease activity of GajA on plasmid DNA. ‘λ955’ is a PCR-amplified DNA fragment. The λ955 linearized fragment is extracted from a plasmid. pUC19-λ955 plasmid is a pUC19 plasmid inserted with the λ955 DNA fragment. pUC19 plasmid was used as a control. GajA activity is efficient on both a PCR fragment and a plasmid DNA containing λ955 sequence. Plasmids were amplified in and extracted from *E. coli* DH5α. “N”, “L”, and “S” mark the positions of “nicked”, “linearized”, and “supercoiled” DNA gel bands, respectively. CK, control reaction without GajA. 125 ng DNA was incubated with 0.2 μM GajA in a final volume of 10 μl in the optimal reaction buffer. Reactions were performed at 37 ℃ for 5 min and then stopped by the addition of 2 μl of 6 × loading dye containing 20 mM EDTA. Samples were analyzed via native agarose gel electrophoresis.

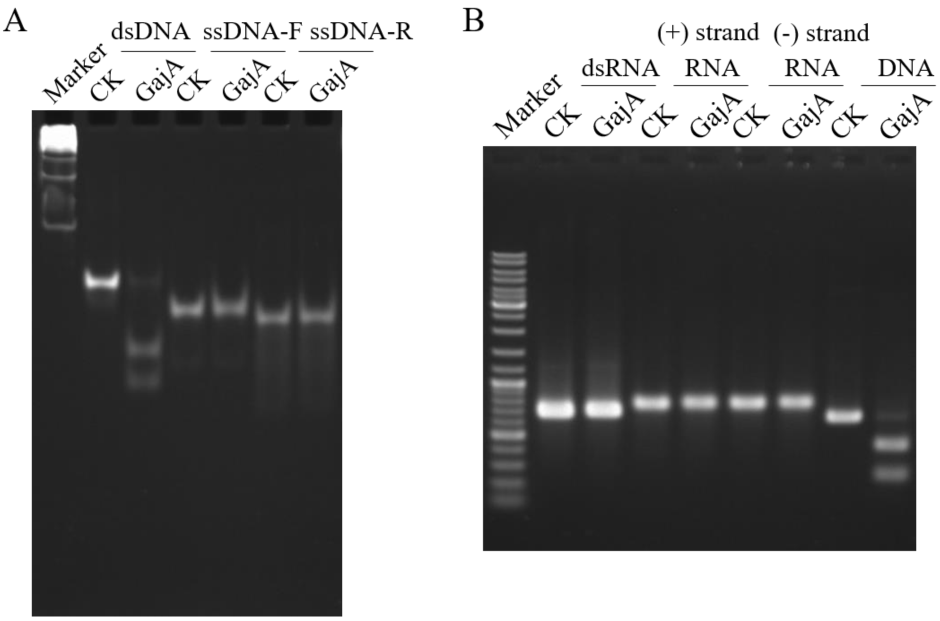

Figure S7. GajA cuts dsDNA specifically. (**A**) GajA exhibits no nuclease activity on ssDNA. dsDNA was prepared by annealing ssDNA-F and ssDNA-R in this assay. (**B**) GajA exhibits no nuclease activity on dsRNA and ssRNA. dsRNA was prepared by annealing (+) strand RNA and (-) strand RNA in this assay. DNA as a control is the transcription template for RNA in this assay. All substrate sequences are listed in Table S5 and all substrates contain the recognition sequence of GajA (underlined in Table S5). CK, control reaction without GajA. The DNAs were incubated with 0.2 μM GajA in a final volume of 10 μl in the optimal reaction buffer. Reactions were performed at 37 ℃ for 5 min and then stopped by the addition of 2 μl of 6× loading dye containing 20 mM EDTA. Samples were analyzed via native agarose gel electrophoresis.

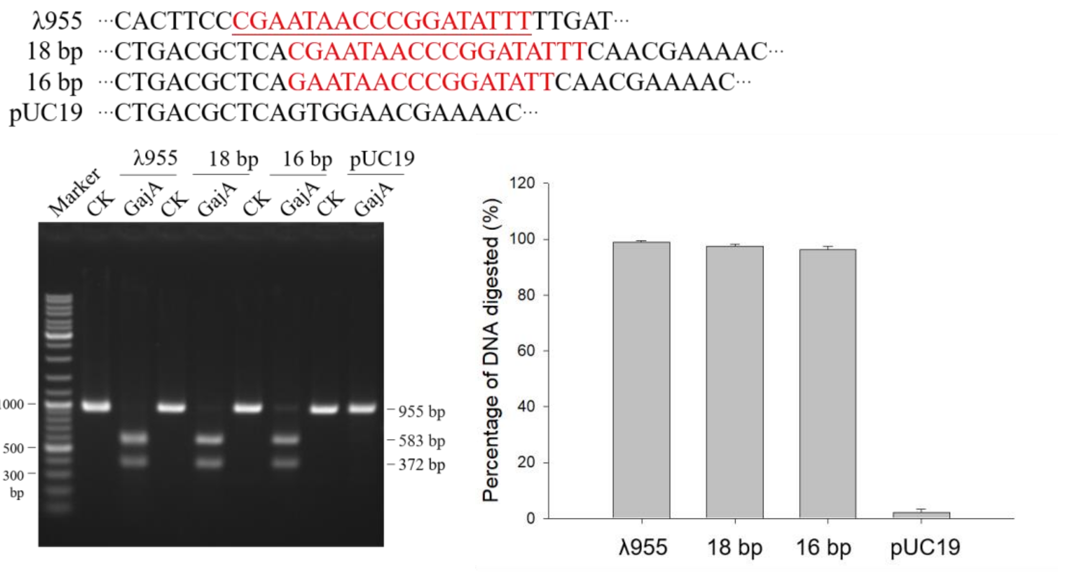

Figure S8. The search for minimum recognition sequence of GajA. DNA sequences from λ955 DNA surrounding the cleavage site of GajA were gradually shortened and inserted into pUC19 plasmid. The 16-bp sequence shown on top of the gel still supported full activity of GajA and was selected as the substrate for further characterization of GajA recognition sequence. In these reactions, 125 ng DNA was incubated with 0.2 µM GajA in a final volume of 10 µl in the optimal reaction buffer. Reactions were performed at 37 ℃ for 5 min and then stopped by the addition of 2 µl of 6× loading dye containing 20 mM EDTA. Samples were analyzed via native agarose gel electrophoresis. All graphs represent the average of three independent trials with error bars representing the standard error of the mean.

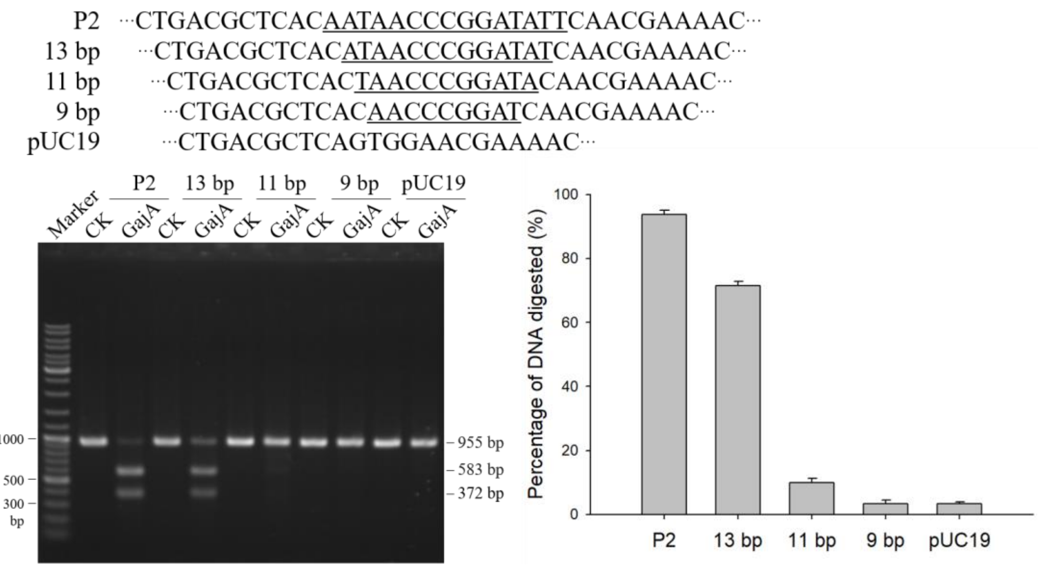

Figure S9. The preliminary recognition sequence of GajA was shortened one by one nucleotide from both ends, and DNA fragments containing the resulting sequences were PCR-amplified as substrates to test GajA cleavage efficiency. DNA digested was quantified using ImageJ software as described in the Materials and Methods. In these reactions, 125 ng DNA was incubated with 0.2 μM GajA in a final volume of 10 μl in the optimal reaction buffer. Reactions were performed at 37 ℃ for 5 min and then stopped by the addition of 2 μl of 6 × loading dye containing 20 mM EDTA. Samples were analyzed via native agarose gel electrophoresis. All graphs represent the average of three independent trials with error bars representing the standard error of the mean.

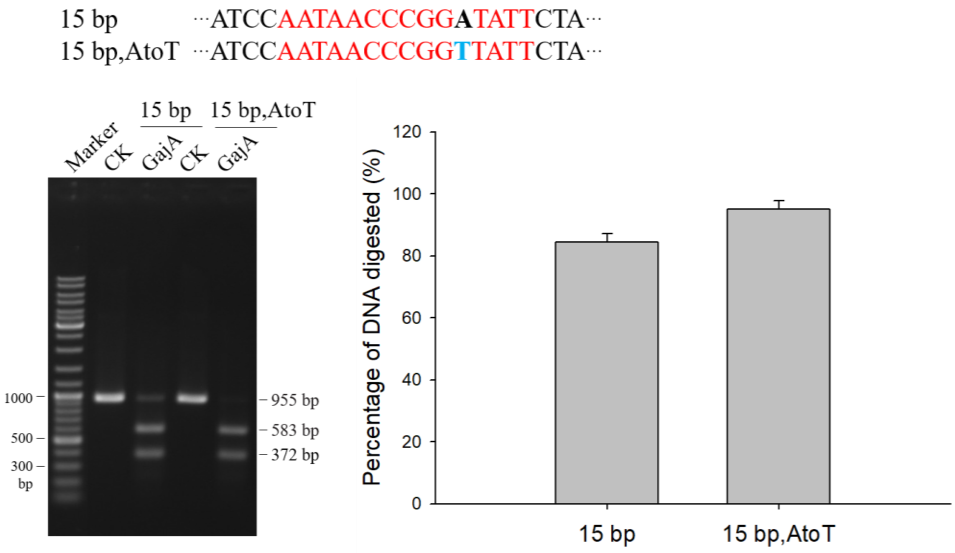

Figure S10. A partial palindrome sequence is the optimal substrate for GajA cleavage. An A-to-T mutation in the 15-bp recognition sequence resulted in a partial palindrome sequence, on which the GajA cleavage was more efficient. 125 ng DNA was used as the substrate and incubated with 0.2 μM GajA in a final volume of 10 μl in the optimal reaction buffer at 37 ℃ for 3 min. Samples were analyzed via native agarose gel electrophoresis. All graphs represent the average of three independent trials with error bars representing the standard error of the mean.

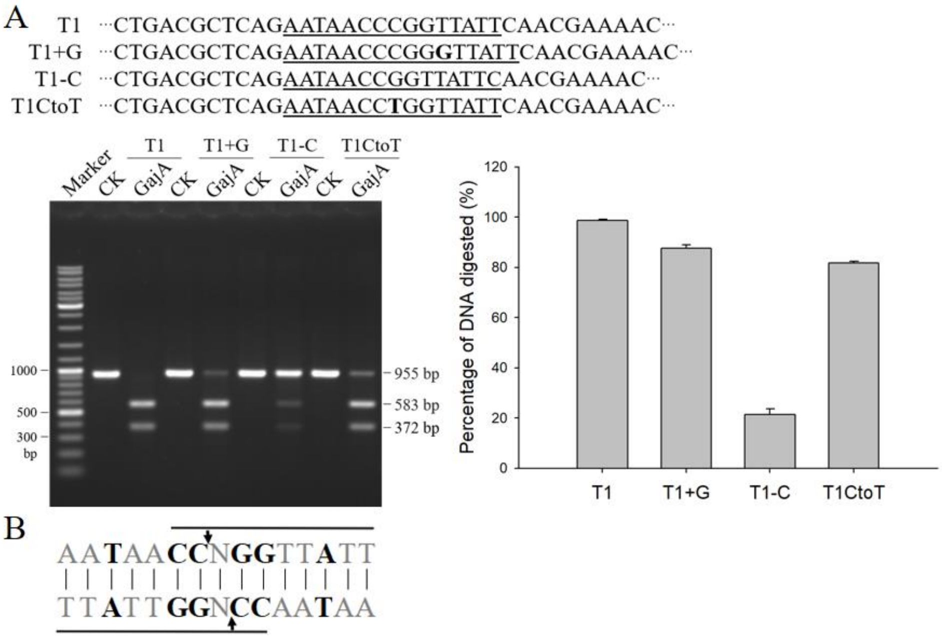

Figure S11. Characterization of the full optimal recognition sequence of GajA. (**A**) Cleavage efficiency (measured as a reduction of initial DNA substrates) of GajA on variety recognition sequences. T1 is the initial λ955 DNA substrate containing the full recognition sequence (underlined) of GajA. T1+G denotes a G (bold) addition into T1 and T1-C denotes a C deletion from T1, which turn the underlined recognition sequences into complete palindromic sequences. T1CtoT represents a T (bold) mutation of the central C in T1 substrate. 125 ng DNA was incubated with 0.2 μM GajA in a final volume of 10 μl in the optimal reaction buffer. Reactions were performed at 37 ℃ for 5 min and then stopped by the addition of 2 μl of 6× loading dye containing 20 mM EDTA. Samples were analyzed via native agarose gel electrophoresis. All graphs represent the average of three independent trials with error bars representing the standard error of the mean. (**B**) The optimal recognition sequence of GajA. The black arrows indicate GajA cleavage sites. Lined sequences represent the minimum recognition sequence. Sequences in black are strict while those in gray are less strict for GajA recognition.

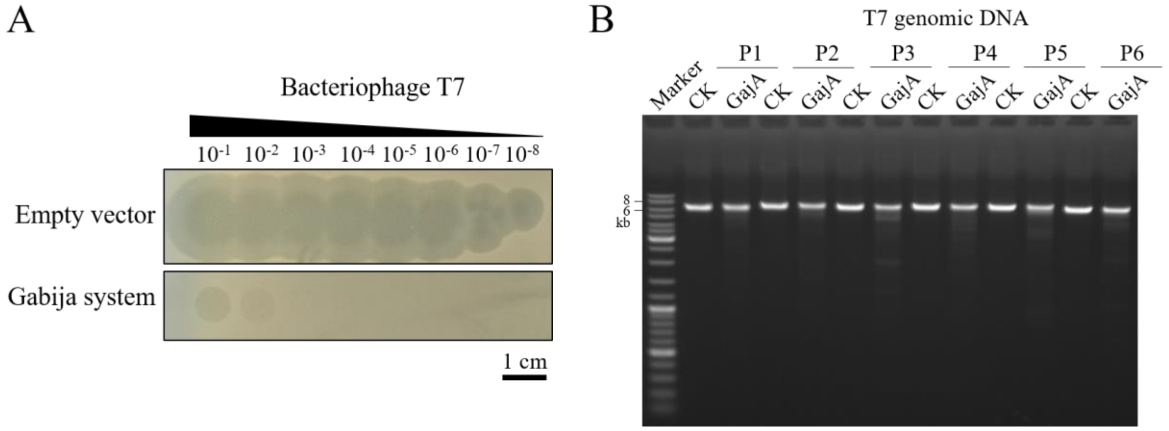

Figure S12. The Gabija system renders *E. coli* resistant to bacteriophage T7, and the GajA cleaves T7 genomic DNA. (**A**) The Gabija system has cross-species function in *E. coli* to abolish bacteriophage T7 infection. The sequences of the Gabija gene cassette including GajA and GajB genes (located at 94190-97412 of the *B. cereus* VD045 genome [AHET01000033]) were cloned into the pQE82L vector. The recombinant vector or empty vector was transformed into *E. coli* B (ATCC® 11303™). A single bacterial colony was picked from a fresh LB agar plate and grown in LB broth containing ampicillin (100 μg/ml) at 37 ℃ to an OD_{600} of ~0.2. The protein expression was induced by the addition of 0.2 mM IPTG. After further growth for 1 h to an OD_{600} of 0.7-0.8, 500 μl of cells were mixed with 14.5 mL of 0.5% LB top agar, and the entire sample was poured onto LB plates containing ampicillin (100 μg/ml) and IPTG (0.1 mM). Plates were spotted with 4 μl of bacteriophage T7 diluted in LB at eight 10-fold dilutions, namely, 10^{-1}-10^{-8}. Plates were incubated at 37 ℃ for 14 h, and then imaged. The scale bar represents 1 cm. (**B**) The six PCR-amplified DNA fragments covering T7 genomic DNA were treated by GajA. P1: 1-6,644 bp; P2: 6,505-13,526 bp; P3: 13,396-20,177 bp; P4: 20,025-26,915 bp; P5: 26,796-33,657 bp; P6: 33,529-39,937 bp. The reactions were carried out as described in Materials and Methods.

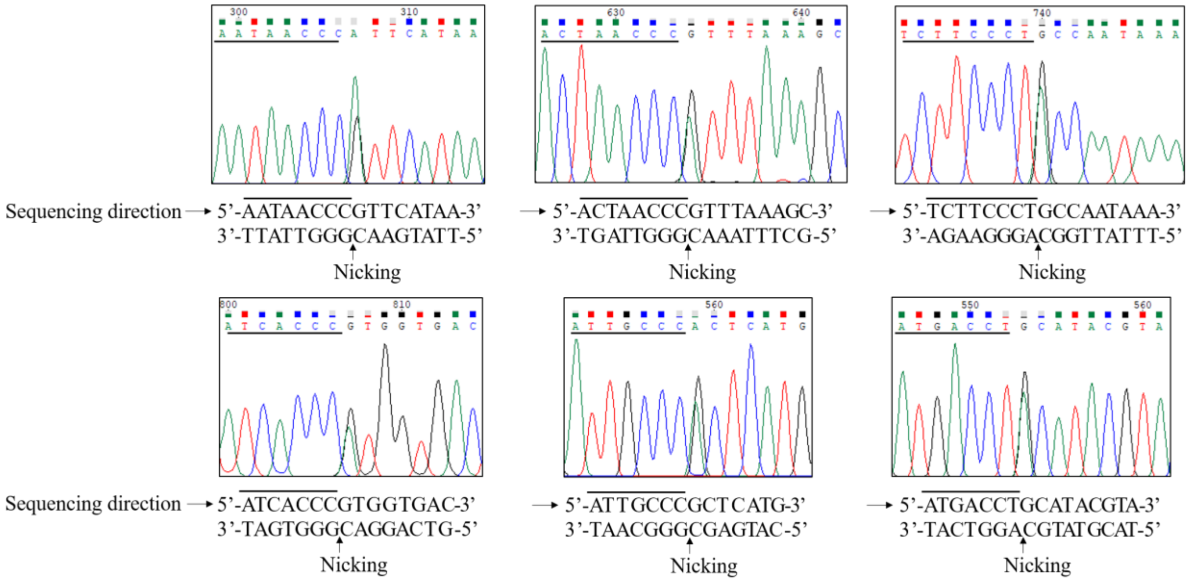

Figure S13. Examples of GajA nicked sites revealed by run-off sequencing. An additional "A" peak indicates a nicked site (denoted by arrows) on the template strands (the extrinsic A was added by Taq DNA polymerase by the template-independent terminal nucleotide transferase activity at the end of the template DNA).

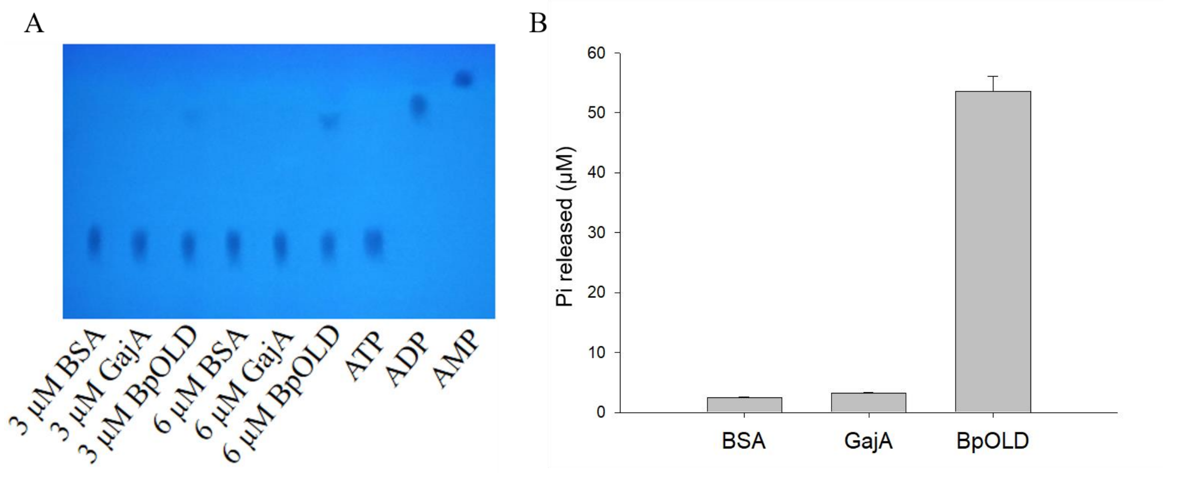

Figure S14. GajA does not have ATPase activity. ATPase activity is detected from BpOLD protein but not GajA by thin layer chromatography (**A**) or the PiColorLock™ phosphate detection system kit (Expedeon) (**B**). The reactions were carried out as described in the Materials and Methods. BSA was used as a control.

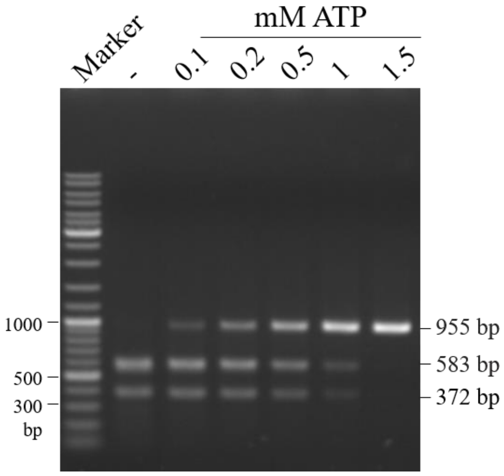

Figure S15. Effect on GajA endonuclease activity by increasing amounts of ATP when the reaction buffer contains 5 mM Mg^{2+}. 125 ng of λ955 DNA (20 nM) was incubated with 0.2 μM GajA in a final volume of 10 μl in the optimal reaction buffer at 37 ℃ for 5 min. Then stopped by the addition of 2 μl of 6× loading dye containing 20 mM EDTA. Samples were analyzed via native agarose gel electrophoresis.

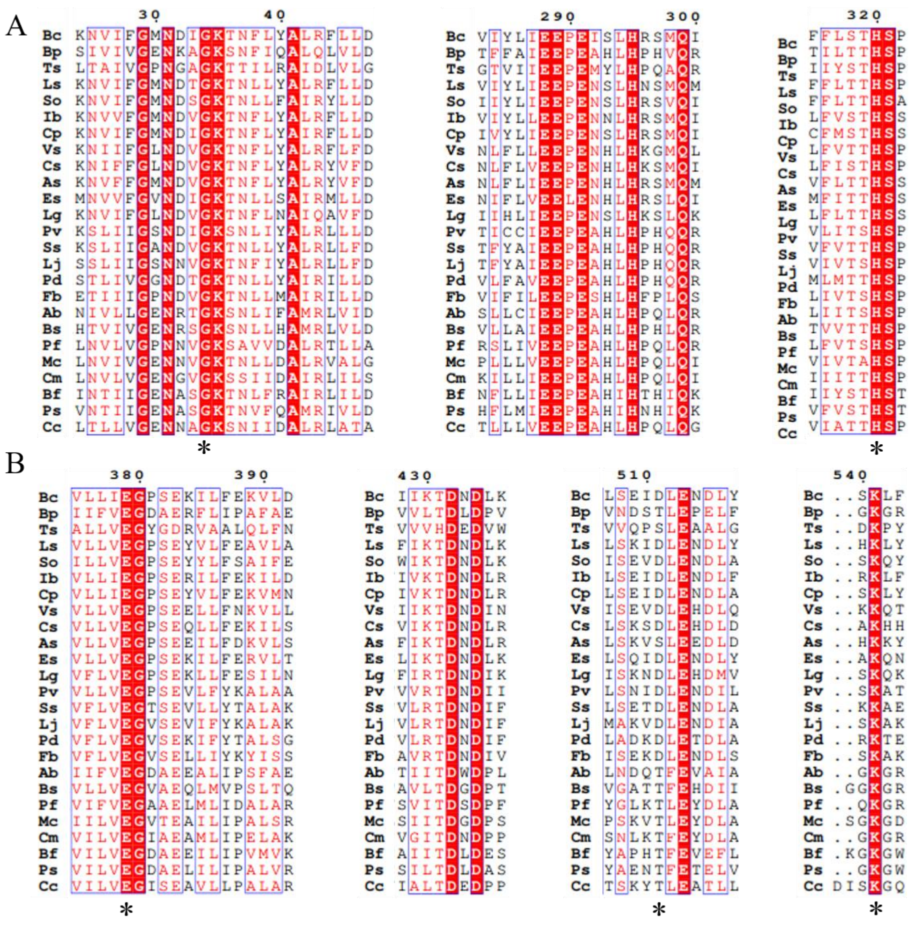

Figure S16. Sequence alignment of potential active sites of GajA and homologs from different species. (**A**) and (**B**) denote the conservative regions of ATPase-like (A) and TOPRIM domain (B), respectively. Asterisks indicate the key residues in the active site verified previously for OLD proteins. 100% conservation is indicated by white text on red background, and 70% conservation by boxed red text on white background. Abbreviations are as following: Bc, *Bacillus cereus* VD045; Bp, *Burkholderia pseudomallei* 668; Ts, *Thermus scotoductus*; Ls, *Leptotrichia* sp. oral taxon 225 str. F0581; So, *Streptococcus oralis*; Ib, *Intestinibacter bartlettii* DSM 16795; Cp, *Clostridium perfringens* C str. JGS1495; Vs, *Veillonella* sp. AS16; Cs, *Clostridium* sp. MSTE9; As, *Acidaminococcus* sp. BV3L6; Es, *Eubacterium* sp. CAG:603; Lg, *Leuconostoc gelidum* subsp. gasicomitatum KG16-1; Pv, *Paenibacillus vortex* V453; Ss, *Shuttleworthia* sp. MSX8B; Lj, *Lactobacillus johnsonii* NCC 533; Pd, *Parascardovia denticolens* IPLA 20019; Fb, *Firmicutes bacterium* CAG:884; Ab, *Acinetobacter baumannii* AB0057; Bs, *Blastococcus saxobsidens*; Pf, *Pseudomonas fluorescens* LBUM223; Mc, *Methanosaeta concilii*; Cm, *Candidatus Methanosphaerula palustris*; Bf, *Bacteroides fragilis* NCTC9343; Ps, *Psychromonas* sp. CNPT3; Cc, *Corynebacterium callunae*.

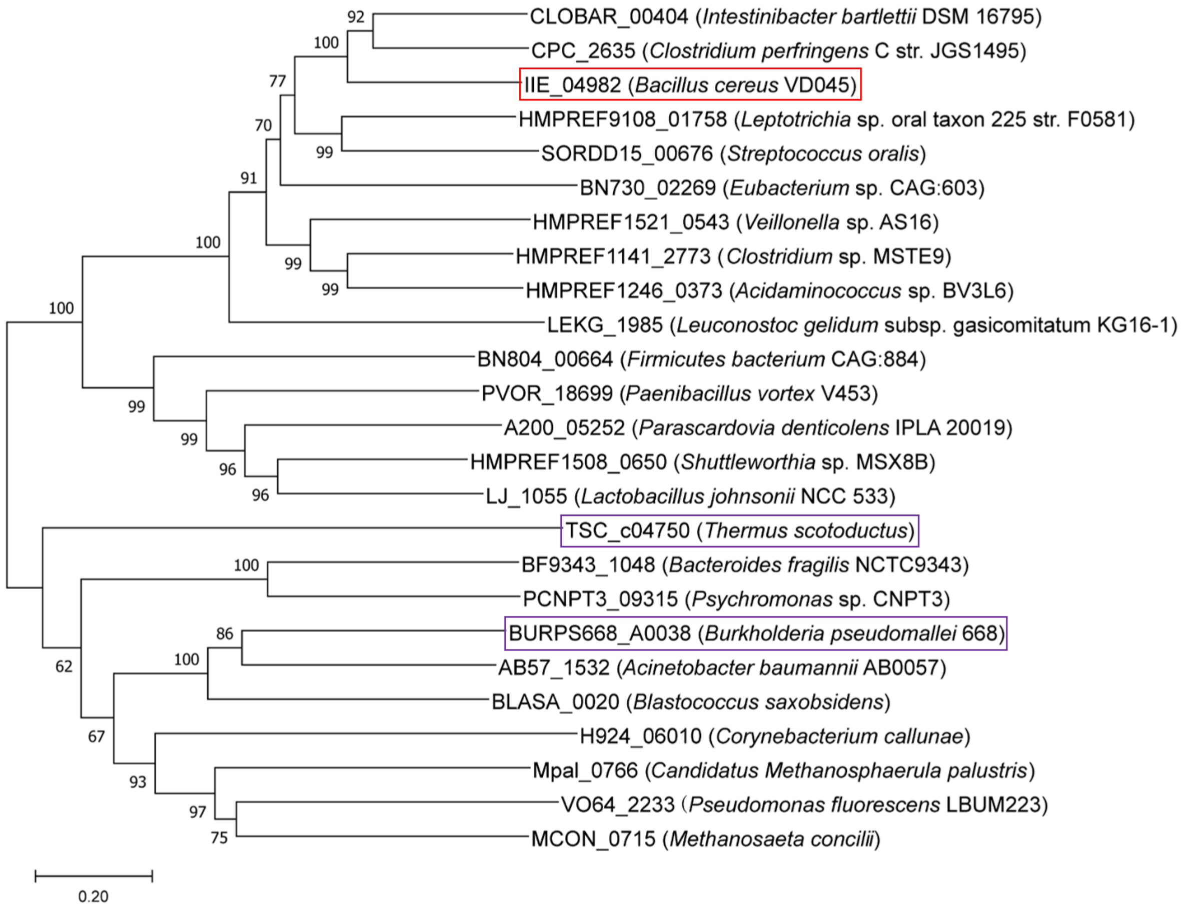

Figure S17. Phylogenetic analysis of GajA and 24 homologs from different species. The numbers on the tree branch are the percentage values after 1000 replications. Branch length represents evolutionary distance. The number of substitutions of amino acids per site is indicated by the scale bar. GajA is marked by a red box. BpOLD and TsOLD are marked by purple boxes.
